# Biological and genomic characteristics of two bacteriophages isolated from sewage, using one multidrug-resistant and one non-multidrug-resistant strain of *Klebsiella pneumoniae*

**DOI:** 10.3389/fmicb.2022.943279

**Published:** 2022-10-13

**Authors:** Bingchun Liang, Wenpeng Zhao, Bo Han, Herman W. Barkema, Yan D. Niu, Yongxia Liu, John P. Kastelic, Jian Gao

**Affiliations:** ^1^Department of Clinical Veterinary Medicine, College of Veterinary Medicine, China Agricultural University, Beijing, China; ^2^Department of Production Animal Health, Faculty of Veterinary Medicine, Hospital Drive NW, University of Calgary, Calgary, AB, Canada; ^3^Department of Ecosystem and Public Health, Faculty of Veterinary Medicine, Hospital Drive NW, University of Calgary, Calgary, AB, Canada; ^4^College of Veterinary Medicine, Shandong Agricultural University, Taian, Shandong, China

**Keywords:** bovine mastitis, *Klebsiella pneumoniae*, bacteriophage, biological characteristics, genomic characteristics

## Abstract

Bovine mastitis caused by multi-drug resistant (MDR) *Klebsiella pneumoniae* is difficult to treat with antibiotics, whereas bacteriophages may be a viable alternative. Our objective was to use 2 *K*. *pneumoniae* strains, 1 MDR and the other non-MDR, to isolate phages from sewage samples and compare their biological and genomic characteristics. Additionally, phage infected mouse mammary gland was also analyzed by H&E staining and ELISA kits to compare morphology and inflammatory factors, respectively. Based on assessments with double agar plates and transmission electron microscopy, phage CM_Kpn_HB132952 had clear plaques surrounded by translucent halos on the bacterial lawn of *K*. *pneumoniae* KPHB132952 and belonged to *Siphoviridae*, whereas phage CM_Kpn_HB143742 formed a clear plaque on the bacterial lawn of *K*. *pneumoniae* KPHB143742 and belonged to *Podoviridae*. In 1-step growth curves, CM_Kpn_HB132952 and CM_Kpn_HB143742 had burst sizes of 0.34 and 0.73 log_10_ PFU/mL, respectively. The former had a latent period of 50 min and an optimal multiplicity of infection (MOI) of 0.01, whereas for the latter, the latent period was 30 min (MOI = 1). Phage CM_Kpn_HB132952 had better thermal and acid–base stability than phage CM_Kpn_HB143742. Additionally, both phages had the same host range rate but different host ranges. Based on Illumina NovaSeq, phages CM_Kpn_HB132952 and CM_Kpn_HB143742 had 140 and 145 predicted genes, respectively. Genomic sequencing and phylogenetic tree analysis indicated that both phages were novel phages belonging to the *Klebsiella* family. Additionally, the histopathological structure and inflammatory factors TNF-α and IL-1β were not significantly different among phage groups and the control group. In conclusion, using 1 MDR and 1 non-MDR strain of *K*. *pneumoniae*, we successfully isolated two phages from the same sewage sample, and demonstrated that they had distinct biological and genomic characteristics.

## Introduction

Bovine mastitis, a common disease on dairy farms worldwide, causes huge economic losses (Angelopoulou et al., 2019). Antibiotics are frequently used to treat bovine mastitis (Klaas and Zadoks, 2018), but overuse has induced multidrug-resistant (MDR) bacterial strains, contributing to treatment failures (Gordillo Altamirano and Barr, 2019). It is well known that *K*. *pneumoniae* is an important cause of bovine mastitis (Gao et al., 2017; Cheng et al., 2020) and many strains of *K*. *pneumoniae* isolated from bovine mastitis are highly resistant to common antibiotics, with various MDR strains detected (Cheng et al., 2019). Phages have much potential as an alternative to antibiotics (Abedon, 2019; Pires et al., 2020). Phages can be used to treat patients infected with multidrug-resistant bacteria, with remarkable therapeutic effects (Corbellino et al., 2020). Phages may be the best agent for treatment of multidrug-resistant bacteria causing bovine mastitis.

Genetic diversity of phages is very high, with an estimated >10^31^ kinds of tailed phage particles in the biosphere (Santos et al., 2018; Dion et al., 2020). Phages are viruses that can lyse bacteria (Röhrig et al., 2020) and based on life cycle, are classified as either lytic or lysogenic (temperate phage). Phages have unique biological properties including a 1-step growth curve, thermal stability and acid–base stability; these biological properties are important indicators for characterizing survival of phages in the external environment. In addition to their biological properties, by exploring phage genomes, we can analyze the genetic characteristics of phages and develop new engineered phages, e.g., an engineered λ phage that enabled enhanced and strain-specific killing of enterohemorrhagic *Escherichia coli* (Jin et al., 2022). Additionally, in an invertebrate *Drosophila melanogaster* model, phage therapy significantly delayed or prevented mortality against a lethal dose of *Pseudomonas aeruginosa* (Melo et al., 2020). In mammals, phage therapy has been effectively used to treat local, gastrointestinal, pulmonary, and systemic infections (Kortright et al., 2019; Corbellino et al., 2020). Phages and their enzyme preparations are ideal alternatives to traditional antimicrobial agents in a world where antimicrobial resistance is emerging and spreading at an unprecedented speed.

Bacteriophages have a high degree of genetic diversity and can carry horizontal gene transfer (e.g., drug resistance genes, virulence genes, etc.) of host bacteria (Salmond and Fineran, 2015). Therefore, their genomic characteristics are closely related to host bacteria. *Klebsiella pneumoniae* is an imporant pathogen causing mastitis in dairy cows; however, whether there are differences in the phage genomic characteristics induced by drug-resistant and non-drug-resistant *K*. *pneumoniae* remains to be clarified. To the best of our knowledge, this is the first study that investigated differences in characteristics of phages induced by 2 strains of *K*. *pneumoniae* (1 MDR and the other non-MDR). This study is expected to provide a meaningful reference for studying *K*. *pneumoniae* phage genome characteristics.

## Materials and methods

### Bacterial strains

In this study, 2 *K*. *pneumoniae* strains (KPHB132952 and KPHB143742) recovered from bovine clinical mastitis (CM) were used to isolate phages from a sewage sample. The 2 strains belonged to the same capsule serotype (K57) and the same clonal pattern based on repetitive element sequence-based PCR. Regarding these 2 strains, KPHB132952 was resistant to ceftiofur-cefquinome-imipenem-enrofloxacin and was therefore defined as an MDR strain. Phage KPHB143742 was only resistant to amoxicillin/clavulanic acid. In addition to KPHB132952 and KPHB143742, 29 *K*. *pneumoniae* strains (9 MDR strains and 20 non-MDR strains, all belonged to K57 capsule serotype), 3 *Escherichia coli* (*E*. *coli*) strains, 1 *Staphylococcus aureus* (*S*. *aureus*) strain, 1 *Streptococcus agalactiae* (*S*. *agalactiae*) strain and 1 *Streptococcus dysgalactiae* (*S*. *dysgalactiae*) strain isolated from CM cows were used to test host range of the two phages. All strains were stored at-80°C. The *K*. *pneumoniae* suspensions were prepared from frozen stocks on Luria-Bertani (LB) nutrient culture medium (Beijing AOBOX Biotechnology Co., Ltd.) and incubated in a shaker (37°C, 220 rpm) for 16 h. The *E*. *coli*, *S*. *aureus*, *S*. *agalactiae*, and *S*. *dysgalactiae* suspensions were prepared from frozen stocks on Brain-Heart Infusion (BHI) nutrient culture medium (Beijing AOBOX Biotechnology Co., Ltd.) and incubated in a shaker (37°C, 220 rpm) for 24 h. Thereafter, *K*. *pneumoniae* strains were cultivated to the logarithmic growth phase (OD_600_ = 0.74) prior to determining MOI, 1-step growth curves, acid–base stability, and thermal stability of phages.

### Bacteriophage isolation and purification

In total, 51 sewage effluent samples were collected from 3 dairy farms near Beijing, China. The 51 samples were collected from cesspools, sewers, drinking ponds, and cesspools at various locations on the farms. Samples (5 ml) were centrifuged at 13,813 × *g* for 10 min and the supernatant was filtered through a 0.22 μm microporous filter (Merck) to obtain the filtrate that was stored at 4°C (~ 2–3 days). The 51 sewage samples were marked from No. 1 to No. 51, then phage isolation was attempted using 2 strains of *K*. *pneumoniae*. For each sewage sample, a mid-log phase broth of the 2 *K*. *pneumoniae* strains (KPHB132952 and KPHB143742) was prepared and 5 ml of liquid LB liquid medium was added after taking 100 μl of phage filtrate (n = 51) and 100 μl of bacterial solution in mid-log phase (n = 2), respectively, with the culture solution shaken (220 rpm) for 20 h at 37°C. The enriched culture was centrifuged at 13,813 × *g* for 10 min at 4°C and the supernatant was filtered through a 0.22 μm microporous filter (Merck) to obtain a bacteriophage stock solution mixture. A mid-log phase bacteria suspension (50 μl), bacteriophage stock solution (50 μl) and LB liquid medium (5 ml) were added into a 10 ml tube. Then, an overlay plaque assay was performed (a double plate was spread) and placed in a 37°C incubator for 1–5 h (optimal time range, based on preliminary test results). After plaque formation, plaques of the same size were picked. Then, 100 μl of the corresponding bacterium host was inoculated into LB liquid medium, and shaken at 37°C overnight (≈16 h). The culture broth was centrifuged 13,813 × *g* for 10 min, the supernatant was filtered through a 0.22 μm microporous filter, and the purified bacteriophage stock solution was stored at 4°C (~ 2–3 days). Purification procedures were conducted as described (Shi et al., 2021) and the titer of the phage inoculum was determined by a plaque assay (Richards et al., 2021). The plaque was examined by a double-layer agar method (Zhao et al., 2021). The *K*. *pneumoniae* bacterium solution in mid-log phase was mixed with a phage stock solution with LB semi-solid medium, and then the double-layer plate was spread evenly and cultured at 37°C to facilitate plaque observation.

### Transmission electron microscopy

The bacteriophage purification solution was diluted with SM solution (comprised of 8 mM magnesium sulfate, 100 mM sodium chloride, and 0.01% gelatin). Purified bacteriophage suspension (log_10_/PFU/mL = 7.66) was stained with 2% uranyl acetate and bacteriophage ultrastructure was observed using TEM (120 KV; HT7800, Hitachi, Beijing, China) in the Laboratory of Electron Microscopy, Faculty of Biology, China Agriculture University, Beijing, China.

### Optimal multiplicity of infection and one-step growth curve of phages

Optimal multiplicity of infection (MOI) of phages titer (PFU/mL)/bacterial suspension (CFU/mL) as 0.001, 0.01, 0.1, 1, 10 and 100 was observed with the double-layer agar plate method (Zhao et al., 2021). Phage and bacterial suspension were mixed 1: 1 for each MOI (50 μl bacteriophage and 50 μl bacteria suspension in mid-log phase) at 37°C for 5 min. The mixture was centrifuged at 2,397 × *g* for 10 min at 4°C and supernatant discarded. The precipitate was resuspended in a centrifuge tube with 5 ml of LB liquid medium and cultured with shaking at 37°C for 2 h. The culture was centrifuged at 13,813 × *g* for 5 min at 4°C and filtered. The supernatant’s titer was determined by a double-layer agar plate, with the highest titer defined as the optimal multiplicity of bacteriophage strain infection.

One-step growth curve was measured under optimal MOI conditions (MOI =1: 1) with a double-layer agar plate (Zhao et al., 2021). Pure phage (50 μl) and bacterium (50 μl) growing in logarithmic growth phase were combined and the mixture was cultured in 20 ml of LB liquid medium at 37°C for 5 min. Thereafter, the culture was centrifuged (13,813 × *g*) for 1 min, and the supernatant resuspended. The centrifuge-resuspend procedure was repeated 3 times. The supernatant was added to 20 ml of LB liquid medium and shaken for 120 min (220 rpm). Subsamples (1.5 ml) were obtained from the tube at 0, 10, 20, 30, 40, 50, 60, 70, 80, 90, 100, 110, and 120 min. After purifying samples (n = 13), a 1-step growth curve was prepared, according to the results of the double-layer plate method.

### pH tolerance and thermal stability of phages

Phage sensitivity to pH and temperature were determined. Acid–base stability was measured by mixing 10 μl of filter-purified phages (log_10_/PFU/mL = 4) with 90 μl of sterile phosphate buffered saline (PBS) with a broad range of pH values (2.0 to 12.0). The phage PBS suspension was incubated at 37°C for 2 h. After doubling the dilution, the phage titer was measured and a pH growth curve prepared. In the thermal stability test, the filter-purified phage (log_10_/PFU/mL = 4) was divided into 5 equal groups. Each group was incubated in a water bath at 30, 40, 50, 60, and 70°C for 90 min, with each test repeated 3 times. Subsamples (1 ml) were collected at every 10 min, centrifuged at 13,813 × *g* for 5 min at 4°C, and supernatant was collected. A double-layer plate was spread *via* the double-layer method incubated in a 37°C constant-temperature incubator for 1 to 5 h (Zhao et al., 2021). Each test was repeated 3 times. Plates were checked and plaque count was based on a plate with a plaque number of ~100. This number was then multiplied by the dilution factor to obtain the plaque forming unit (PFU) titer per 1 ml of phages. After calculating the number of plaques, thermal stability curves were prepared.

### Host range analysis

The host range was established by spot testing (Richards et al., 2021). Fresh solutions of *K*. *pneumoniae* or *E*. *coli* (~200 μl) were applied to the entire LB petri dish with a coating stick. In addition, fresh solutions (~ 200 μl) of *S*. *aureus*, *S*. *agalactiae*, or *S*. *dysgalactiae* was also applied to the 5% sheep blood agar plate with a coating stick. Then, 5 μl of phage suspension was spotted onto each bacterial lawn. After incubation overnight (16 h) at 37°C, plates were checked for clear plaques. Results were described in 2 groups: clear zones (+) or no plaques (−).

### Phage DNA extraction and sequencing

Genomic DNA of bacteriophages was isolated from cell pellets with a TIANamp Virus DNA/RNA Kit (Tiangen Biotech Co., Ltd., Beijing, China) according to manufacturer’s instructions. Sequencing was done by Shanghai Biozeron Biotechnology Co., Ltd. (Shanghai, China). The qualified Illumina (150 paired-end reads) library was used for Illumina NovaSeq 6,000 sequencing (Shanghai Biozeron Co., Ltd).

### Genome bioinformatics analyses

The raw paired end reads were trimmed and quality controlled by Trimmomatic with parameters (SLIDINGWINDOW:4:15 MINLEN:75) (Version 0.36).^1^ Clean data derived after implementing the above-described quality control processes were used to conduct further analyses. First, ABySS^2^ was used for genome assembly with multiple-Kmer parameters and optimization of the assembly. Thereafter, GapCloser software^3^ was used to fill residual local inner gaps and correct single-base polymorphism for final assembly.

Gene models were identified using GeneMark and all gene models were compared (blastp) against non-redundant (NR) genes in the NCBI database, SwissProt,^4^ Kyoto Encyclopedia of Genes and Genomes (KEGG),^5^ Clusters of Orthologous Groups of proteins (COG),^6^ and Gene Ontology (GO)^7^ to enable functional annotation by the blastp module. Sequence read archive (SRA) accession numbers were: SRR19174839 and SRR19174838. Additionally, on the basis of homologous gene analysis, we selected the homologous genes that all the species involved in the analysis contained and had only a single copy (to avoid interference of paralogous proteins), and we used Mafft software to perform multiple sequence alignment of these homologous genes. All aligned homologous genes were concatenated to obtain the alignment results at the whole genome level, and then the phylogenetic tree was constructed using Fasttree software.

### Phage-infected mouse mammary gland model

Nine ICR mice (21 days of pregnancy) were housed in the laboratory animal room at the Experimental Animal Center of China Agricultural University. Animal experiments were conducted following protocols approved by the Animal Care and Use Committee (IACUC) of China Agricultural University, Beijing. Phage CM_Kpn_HB132952 and CM_Kpn_HB143742 were used to establish the experimental phage model by being injected into the mammary gland through the distal end of the nipple. Mice were broadly divided into 3 major groups as follows: Control group; phage CM_Kpn_HB132952 group (infection 10^9^ PFU/mL); and phage CM_Kpn_HB143742 group (infection 10^9^ PFU/mL). Three mice from each group were euthanized 24 h post-infection. Mammary gland tissue from each mouse was removed aseptically and fixed in 4% paraformaldehyde for 48 h. The fixed specimen was processed by conventional methods, including dehydration, paraffin embedding and sectioning, and staining with hematoxylin and eosin (H&E). To detect cytokines, mammary gland tissue was homogenized using a homogenizer and centrifuged 13,813 × *g* for 10 min. Concentrations of TNF-α and IL-1β in the supernatant were determined with ELISA kits (Enzyme-linked Biotechnology Co., Ltd., Shanghai, China), in accordance with manufacturer’s instructions.

### Statistical analyses

All data were expressed as mean ± standard deviation and assessed by SPSS 22.0 (SPSS Inc., Chicago, IL, United States). One-way ANOVA was used to compare, among groups, protein expression of inflammatory factors in mammary gland tissue. For all analyses, *p* < 0.05 was considered significant.

## Results

### Plaques and ultrastructures

**Figure 1:**
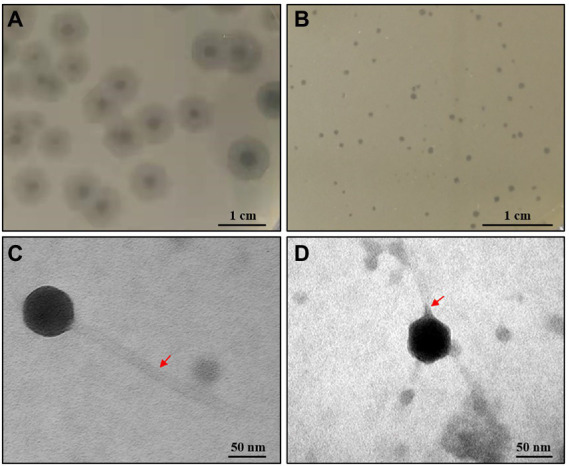
Plaques and ultrastructure observations of phages. **(A,B)** Plaques formed of phage CM_Kpn_HB132952 and CM_Kpn_HB143742 on the lawn of KPHB132952 and KPHB143742, respectively. **(C,D)** Morphologies of bacteriophage CM_Kpn_HB132952 and CM_Kpn_HB143742. The red arrow points to the tail of the phage.

In this study, two phages (CM_Kpn_HB132952 and CM_Kpn_HB143742) were isolated from the same sewage sample (No. 23) using 2 *K*. *pneumoniae* strains (KPHB132952 and KPHB143742). Phage CM_Kpn_HB132952 formed clear plaques surrounded by translucent halos (diameter, 0.85 cm; Figure 1A), whereas phage CM_Kpn_HB143742 formed small clear plaques (diameter, 0.5–1 mm; Figure 1B) on the lawn of KPHB132952 and KPHB143742 host, respectively. Electron microscopy studies revealed that phage CM_Kpn_HB132952 (diameter, 69.1 nm; Figure 1C) had a long tail (281.9 nm) with tail fiber, whereas phage CM_Kpn_HB143742 (diameter, 61.3 nm; Figure 1D) had a short tail (10.5 nm) with tail fiber.

### Biological properties

**Figure 2:**
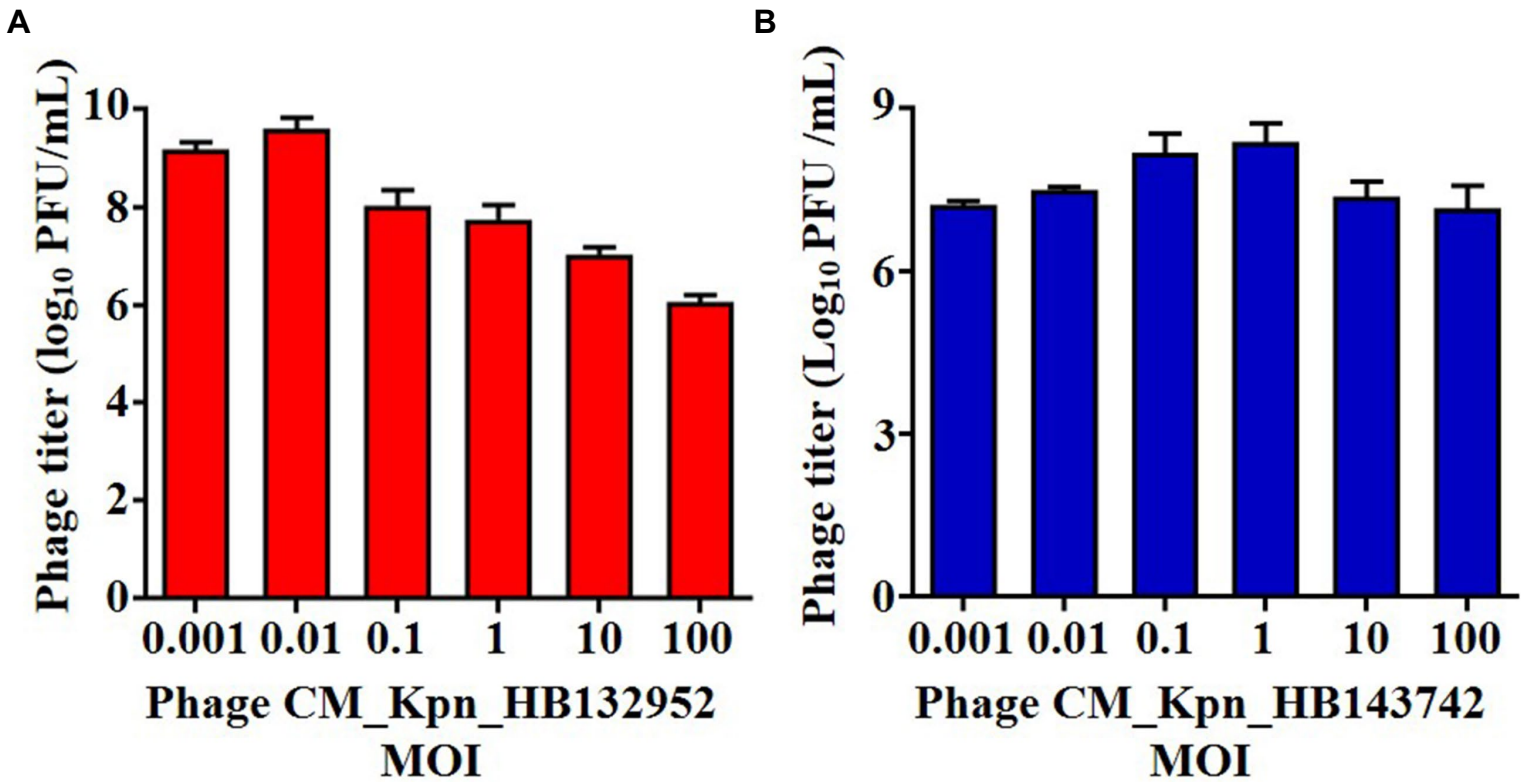
Optimal multiplicity of infection measurements of phages. **(A,B)** Optimal multiplicity of infection of phage CM_Kpn_HB132952 and CM_Kpn_HB143742, respectively. Data represent Mean ± SD of three biological experiments.

**Figure 3:**
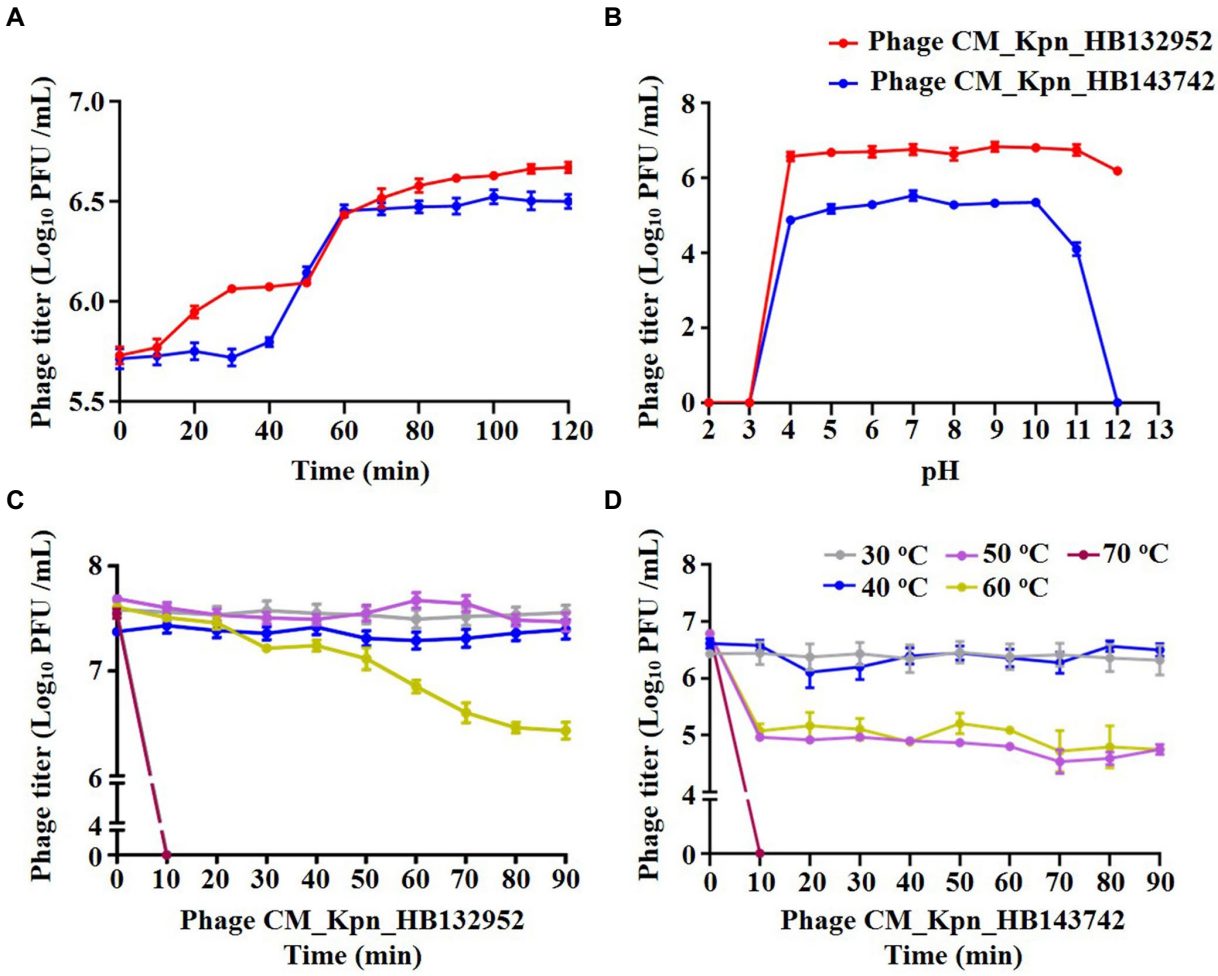
Biological property analyses of phages. **(A)** One-step growth curve of phage CM_Kpn_HB132952 (red) and CM_Kpn_HB143742. **(B)** Acid–base stability of phage CM_Kpn_HB132952 (red) and phage CM_Kpn_HB143742 (blue). **(C,D)** Thermal stability of phage CM_Kpn_HB132952 and phage CM_Kpn_HB143742, respectively. Data represent Mean ± SD of three biological experiments.

The optimal MOI of phage CM_Kpn_HB132952 was 0.01 (ratio of phage to bacteria was 1:100), resulting in the highest phage titer (Figure 2A). The optimal MOI of phage CM_Kpn_HB143742 was 1 (ratio of phage to bacteria was 1:1), resulting in the highest phage titer (Figure 2B). In 1-step growth curves, latency periods of phages CM_Kpn_HB132952 and CM_Kpn_HB143742 lasted ~50 and 30 min, respectively, at 37°C. The average burst size of phages CM_Kpn_HB132952 and CM_Kpn_HB143742, were ~ 0.34 and 0.73 log_10_ PFU /mL progeny phage per cell. During the plateau period (60 to 120 min), there was no significant change in number of phages (Figure 3A). Phage CM_Kpn_HB132952 became inactivated under extreme conditions (pH ≤ 3.0; temperature of 70°C), whereas phage CM_Kpn_HB143742 was inactivated in pH ≤ 3.0, pH ≥ 12.0, or temperature of 70°C. They had different indicators of activated performance (Figure 3B); in that regard, CM_Kpn_HB143742 (Figure 3C) was more sensitive to heat than CM_Kpn_HB132952 (Figure 3D), with the titer decreased nearly 1.76 log_10_ PFU/mL progeny phage per cell at 50 and 60°C.

### Host range analyses

Both phages CM_Kpn_HB132952 and CM_Kpn_HB143742 had broad host ranges (30, 97%) within the 31 *K*. *pneumoniae* strains tested (Table 1). Both phages were able to lyse each other’s separated strains. However, phages had larger and more transparent plaques when lysing the strain from which they were isolated.

**Table 1:** Lytic activity of phages CM_Kpn_HB132952 and CM_Kpn_HB143742 against tested CM strains.

Strain ID	CM_Kpn_HB132952	CM_Kpn_HB143742	Strain ID	CM_Kpn_HB132952	CM_Kpn_HB143742
KPHB2018S28	+	+	KPHB132955	+	+
KPHB6133	+	+	KPHB155141	+	+
KPHB163294	+	+	KPHB143352	+	+
KPHB155643	+	+	KPHB163877	+	+
KPHB155060	+	+	KPHB121006	+	+
KPHB13381	+	+	KPHB154672	+	+
KPHB132732	−	+	KPHB154732	+	+
KPHB154724	+	+	KPHB120296	+	+
KPHB10949	+	+	KPHB154879	+	+
KPHB132952^*^	+	+	KPHB132662	+	+
KPHB143742^*^	+	+	KPHB120094	+	+
KPHB16141	+	+	KPHB132463	+	+
KPHB163287	+	+	KPHB120135	+	+
KPHB132688	+	+	KPHB163356	+	+
KPHB154955	+	+	KPHB155193	+	−
KPHB143397	+	+	*E*. *coli*-1	−	−
*E*. *coli*-2	−	−	*E*. *coli*-3	−	−
*S*. *aureus*	−	−	*S*. *agalactiae*	−	−
*S*. *dysgalactiae*	−	−			

(+) clear zones or (−) no plaques after infection of tested bacteria with both phages. KPHB132952 and KPHB143742 strains were used to induce phage CM_Kpn_HB132952 and CM_Kpn_HB143742, and marked with an asterisk (*) in the upper right corner.

### Whole-genome analyses

**Figure 4:**
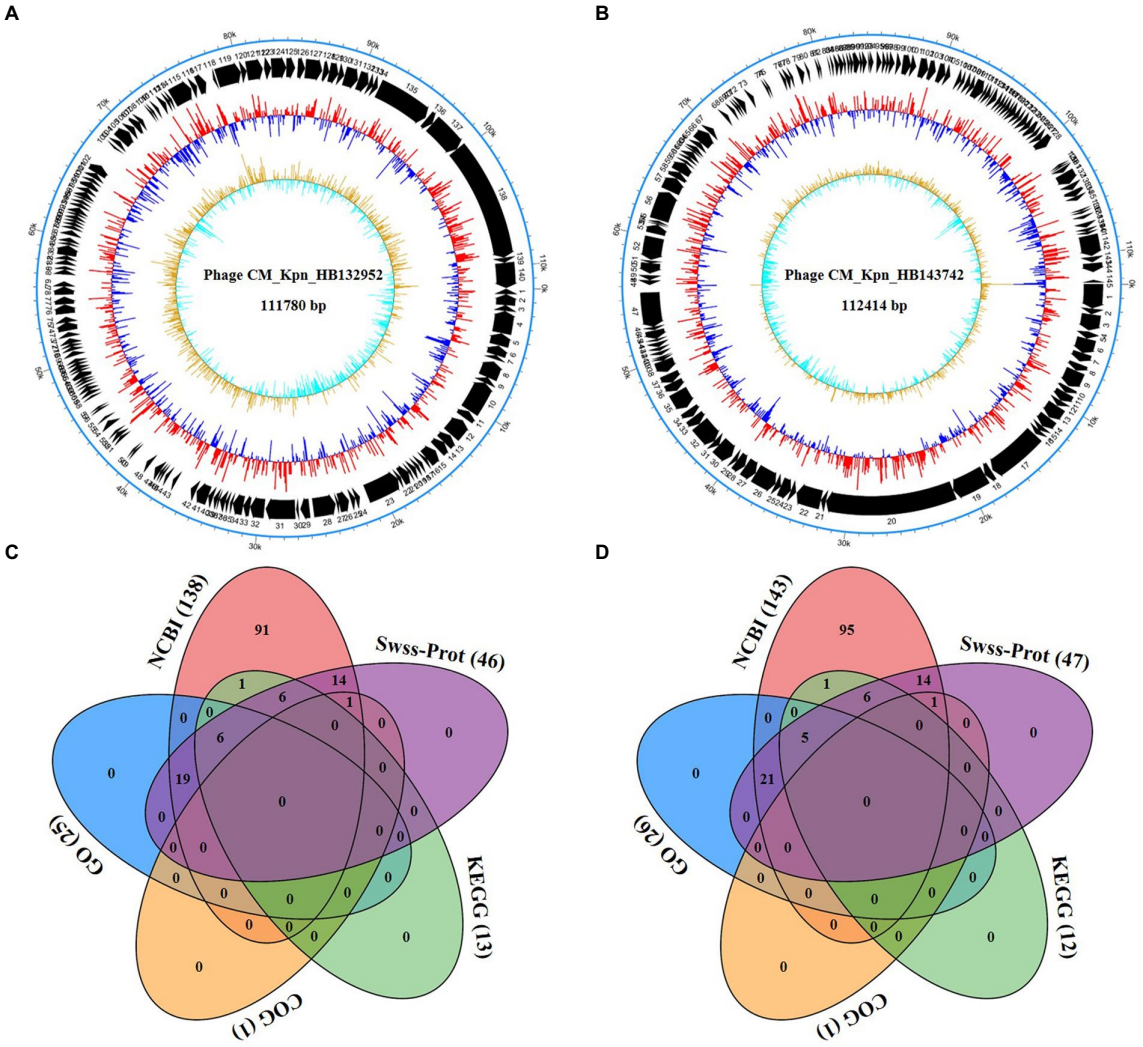
Whole-genome analyses of phages. **(A,B)** The genome structure of phage CM_Kpn_HB132952 and CM_Kpn_HB143742, respectively. **(C,D)** Predicted gene annotation numbers in NCBI, Swiss-Prot, GO, COG, and KEGG databases of phage CM_Kpn_HB132952 and CM_Kpn_HB143742, respectively.

**Figure 5:**
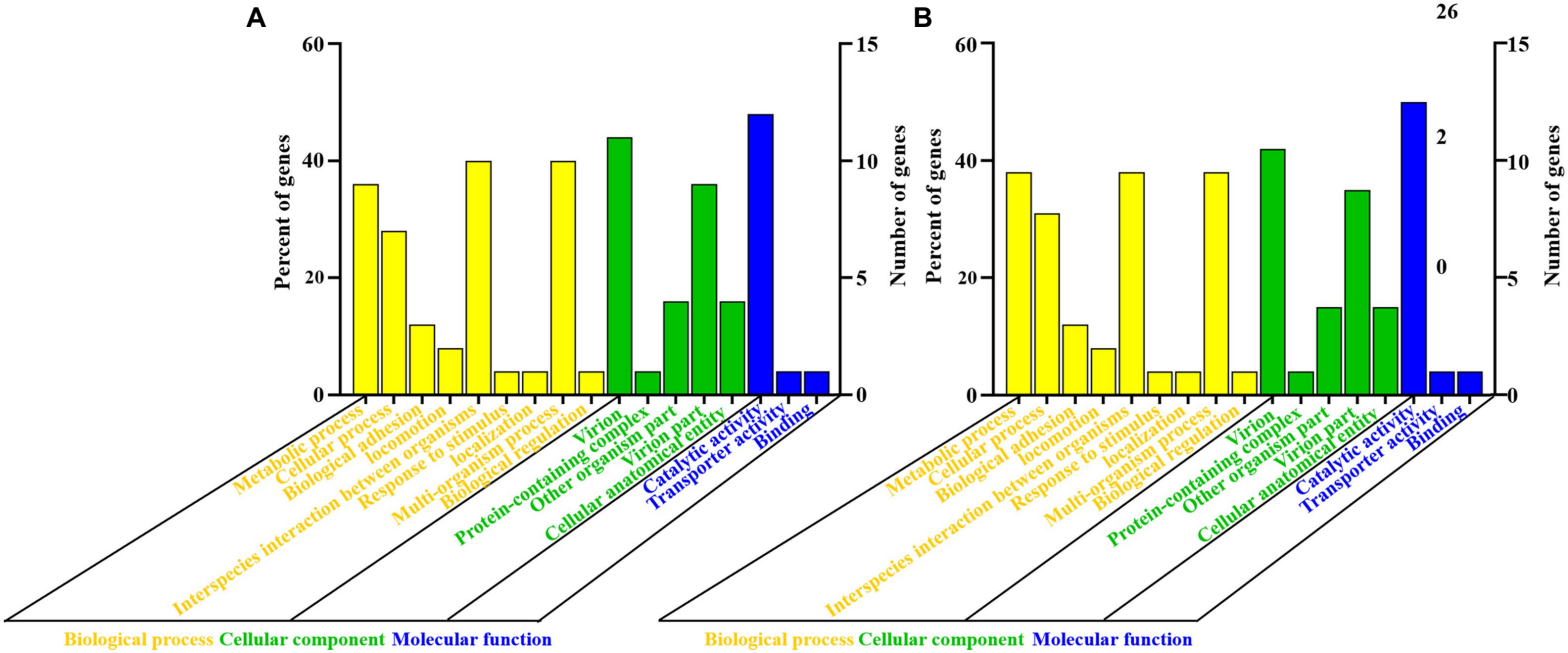
Gene ontology functional annotation of phages. **(A,B)** Gene ontology (GO) analysis of phage CM_Kpn_HB132952 and CM_Kpn_HB143742, respectively. Based on GO functional annotation, phage CM_Kpn_HB132952 had a total of 25 genes and CM_Kpn_HB143742 had a total of 26 genes.

Genomic structures of phages (CM_Kpn_HB132952 and CM_Kpn_HB143742) are shown (Figures 4A,B). Predicted gene information of phages is shown in Table 2. The two phages had distinct genomic characteristics, with sequencing of phage CM_Kpn_HB132952 had 111,780 bp, with a GC content of 45.38%, whereas phage CM_Kpn_HB143742 had 112,414 bp, with a GC content of 45.39%. Phage CM_Kpn_HB132952 and CM_Kpn_HB143742 had total predicted gene numbers of 140 and 145, respectively. Gene total length of phage CM_Kpn_HB132952 and CM_Kpn_HB143742 was 95,178 and 96,279, respectively. Gene average length of phage CM_Kpn_HB132952 and CM_Kpn_HB143742 was 679 and 663. Gene density (genes per 1,000 bp) was 1.252 and 1.289, respectively. The GC content in the gene region of both two phages was 45.8. Plus/minus gene No. of phage CM_Kpn_HB132952 and CM_Kpn_HB143742 was 109/31 and 111/34, respectively. All predicted genes annotation information in NCBI, KEGG, COG and GO databases of phage CM_Kpn_HB132952 and CM_Kpn_HB143742 were analysed and compared to phage CM_Kpn_HB143742; note that different predicted genes of phage CM_Kpn_HB132952 are in red. Compared to phage CM_Kpn_HB132952, different predicted genes of phage CM_Kpn_HB143742 are in blue (Supplementary Tables S1–S4). The sequence of phage CM_Kpn_HB132952 was compared against the NCBI, Swiss-Prot, GO, COG and KEGG databases, yielding 138, 46, 25, 1, and 13 predictive gene annotation information results, respectively (Figure 4C). Furthermore, phage CM_Kpn_HB143742 had 143, 47, 26, 1, and 12 predicted annotation information results in the 5 databases, respectively (Figure 4D). Based on GO functional annotation, phage CM_Kpn_HB132952 had a total of 25 genes, including 43 genes involved in biological process, 29 genes in cellular component and 14 genes in molecular function (Figure 5A). CM_Kpn_HB143742 had a total of 26 genes, including 46 genes involved in biological process, 29 genes in cellular component and 15 genes in molecular function (Figure 5B).

**Table 2:** Predicted gene information of phages CM_Kpn_HB132952 and CM_Kpn_HB143742.

Sample ID	CM_Kpn_HB132952	CM_Kpn_HB143742
Sequence No.	1	1
Total length (bp)	111,780	112,414
GC Content (%)	45.38	45.39
N rate (%)	0	0
Gene No.	140	145
Gene total length (bp)	95,178	96,279
Gene average length (bp)	679	663
Gene density (genes per 1,000 bp)	1.252	1.289
GC content in gene region (%)	45.8	45.8
Gene/genome (%)	85.1	85.6
Plus gene No.	109	111
Minus gene No.	31	34

**Figure 6:**
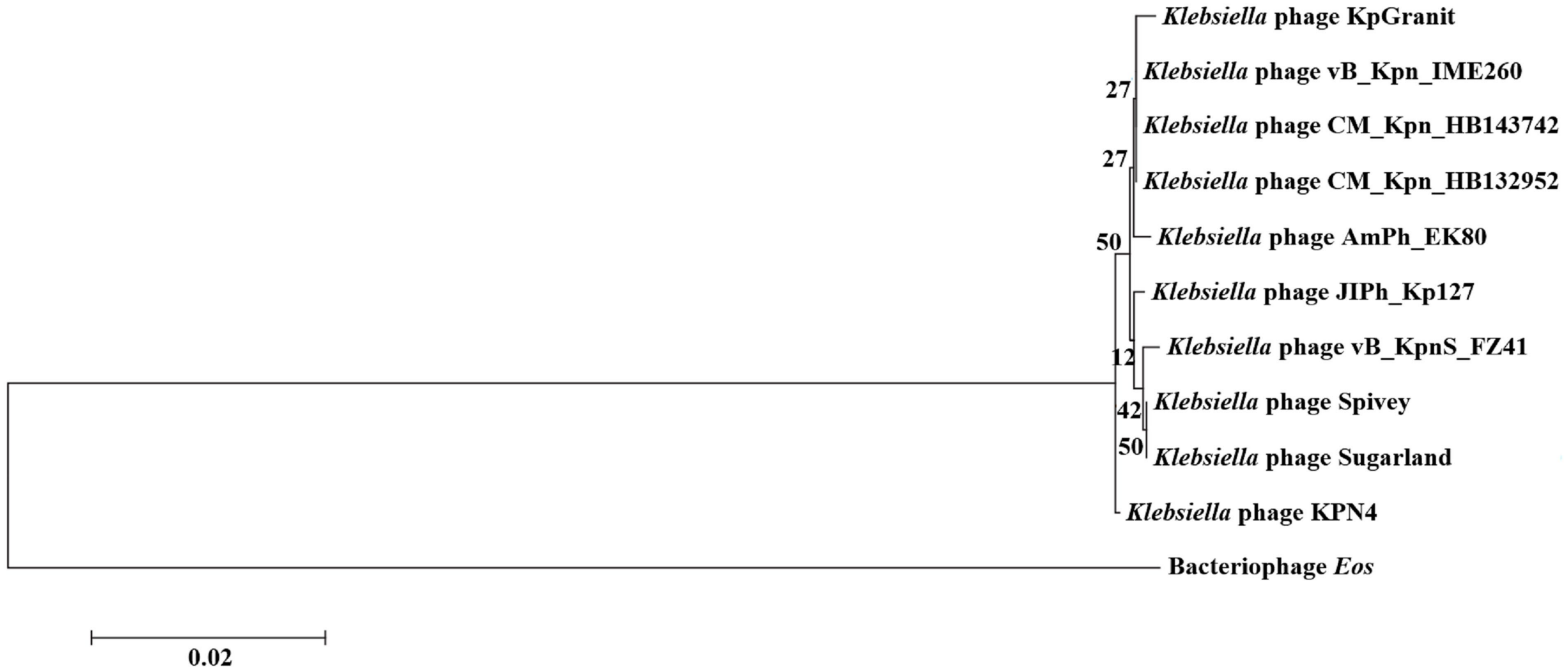
Phylogenetic relationships of phage CM_Kpn_HB132952 and CM_Kpn_HB143742. Phylogenetic tree of phage CM_Kpn_HB132952 and CM_Kpn_HB143742 constructed based on DNA polymerase. The horizontal direction represents the degree of change of genetic variables, and the branches in the horizontal direction represent the changes of evolutionary lineages over time. The longer the branch length, the greater the change of the corresponding species of the branch. The scale is equal to 0.02, indicating that a branch of this length represents a genetic variability of 0.02 for the genome. Branch lengths represent gene sequence similarity. The number on the branch represents the Bootstrap value for that node.

Both phages had greater genetic similarity to *Klebsiella* phage JIPh_Kp127 (CM_Kpn_HB132952 or CM_Kpn_HB143742 = 25/25%), but lesser genetic similarity to *Klebsiella* phage vB_KpnS_FZ41 (CM_Kpn_HB132952 or CM_Kpn_HB143742 = 1/2%) (Table 3). Based on the phylogenetic tree, the closet relatives of phage CM_Kpn_HB132952 and CM_Kpn_HB143742 were vB_Kpn_IME260 (Figure 6). In addition, these two phages each had two genes without corresponding annotation information in each database.

**Table 3:** Comparison of phage CM_Kpn_HB132952 and CM_Kpn_HB143742 predicted genes with genes in the NCBI database.

Phage name (NCBI)	CM_Kpn_HB132952 HSGS^a^ (TRG^b^)	CM_Kpn_HB143742 HSGS^a^ (TRG^b^)
*Klebsiella* phage vB_Kpn_IME260	17 (24)	17 (25)
*Klebsiella* phage Sugarland	14 (19)	14 (20)
*Klebsiella* phage Spivey	8 (11)	6 (9)
*Klebsiella* phage vB_KpnS_FZ41	1 (2)	2 (3)
*Klebsiella* phage KpGranit	9 (12)	8 (11)
*Klebsiella* phage KPN4	12 (16)	12 (17)
*Klebsiella* phage AmPh_EK80	14 (19)	15 (22)
*Klebsiella* phage JIPh_Kp127	25 (35)	25 (36)

a HSGS indicates high similarity gene segments.

b TRG indicates total reference genes.

### Predicted protein analyses

Phage CM_Kpn_HB132952 contained 137 predicted proteins, and phage CM_Kpn_HB143742 had 143 total predicted proteins. Among them, there were a total of 125 identical proteins, but 12 and 18 (phages CM_Kpn_HB132952 and CM_Kpn_HB143742) predicted proteins were different. Differences between the two phages were in hypothetical protein, endonuclease, ribonucleotide reductase, deacetylase, DNA polymerase, holin, tail protein, and tail fiber proteins (Supplementary Table S5). The two phages had the same predicted protein of DNA polymerase, hypothetical protein, and DNA helicase with different gene lengths. Furthermore, identical predicted DNA helicase of phage CM_Kpn_HB132952 was codified by genes 8 and 93, but with different gene lengths.

The two phages shared a total of 48 different predicted proteins, including 9 enzymes (ligase, polymerase, hydrolase, phosphatase, reductase, helicase, endonuclease, deacetylase, and ribonuclease,), 4 structural proteins (transport protein, tail protein, head protein, capsid protein), 2 cleavage-associated protein (holin and endolysin) and 1 other (subunit). Furthermore, both phages encoded their own DNA polymerases and lysis cassette composed of holin and endolysin. Although not conclusive, that plaque halos of phage CM_Kpn_HB132952 increased in size implied the presence of functional depolymerases not apparent in annotation information. It is noteworthy that there were still 65% (100) predicted proteins with unknown functions (Supplementary Table S5).

### Histopathological observations and inflammatory factor analyses

Regarding histopathological observations, all murine mammary gland tissues were white but not swollen (Supplementary Figures S1A1-C1), and there were no differences between phages (CM_Kpn_HB132952 and CM_Kpn_HB143742) group and control group (Supplementary Figures S1A2-C2) with regards to exfoliated mammary epithelial cells or inflammatory infiltrates in mouse mammary acini. Additionally, the content of inflammatory factors TNF-α and IL-1β in murine mammary glands tissue was not different (*p* > 0.05) among phages groups and control group (Supplementary Figures S1D,E).

## Discussion

In this study, we successfully isolated 2 *K*. *pneumoniae* phages CM_Kpn_HB132952 and CM_Kpn_HB143742 from a sewage sample. Phage CM_Kpn_HB132952 belonged to *Siphoviridae* and had a long and non-contractile tail, whereas phage CM_Kpn_HB143742 belonged to *Podoviridae* and had a short tail (Dion et al., 2020). These two phages had differences in MOI, 1-step growth curve, acid–base stability, thermal stability, and host range. In addition, both two phages had distinct numbers of bases, GC content, plus gene number, and minus gene number; however, they had no significant differences regarding their effects on murine mammary tissue morphology or inflammatory factors.

Phage CM_Kpn_HB132952 differed in tail length from previously isolated long-tailed *K*. *pneumoniae* phages CM8-1 and SJT-2 (Shi et al., 2021; Zhao et al., 2021). Furthermore, CM_Kpn_HB143742 had different plaque characteristics than *K*. *pneumoniae* phages πVLC1, πVLC2, πVLC3, and πVLC4 of *Podoviridae* (Domingo-Calap et al., 2020). Phage CM_Kpn_HB132952 was more tolerant to both strong base and heat than phage CM_Kpn_HB143742. Failure of these phages to survive under low or high pH or high temperature conditions would seriously limit direct clinical applications. However, a therapeutic phage can be protected in polymeric microparticles, liposomes, or other forms of encapsulation that can improve antibacterial activity *in vivo* compared to free phage (Dabrowska, 2019). Advances in synthetic biology and phase engineering to engineer phages, including development of temperate phages, could successfully improve their clinical coverage, potency and ability to eradicate bacterial pathogens (Nale and Clokie, 2021).

Host range and bacteriostasis efficacy of phage are closely related to the effects of phage therapy (Dabrowska and Abedon, 2019). In addition, host range is closely associated with its related functional protein, which can be broadened through genome technology (Hesse and Adhya, 2019). In this study, host range results indicated that both phages had broad activity against clinical *K*. *pneumoniae* isolates, but could not lyse clinical *E*. *coli*, *S*. *aureus*, *S*. *agalactiae*, or *S*. *dysgalactiae* isolates. There were differences in the *K*. *pneumoniae* that the two phages can lyse, but the same host range. Although the two phages were able to lyse each other’s separated strains, lysis effects on its separated strain were better. According to this section, a single phage had a limited host range, although this can be addressed through phage cocktails and engineered phages.

Whole-genome sequencing facilitates development of novel treatments (Houldcroft et al., 2017). In this study, both phages CM_Kpn_HB132952 and CM_Kpn_HB143742 belonged to *Klebsiella* phage. These 2 phages had distinct predicted tail protein, tail fiber protein, flap endonuclease, holin, with differences in plaques, morphological structures, biological characteristics, and host range. Different tail proteins may indicate that they had different polysaccharide depolymerase, with a resulting difference in plaques. In addition, phages use the ribosomes of host bacteria to synthesize progeny phages. Development of cellular metabolism, DNA replication and repair, and protein synthesis are affected by NAD-dependent protein deacetylase, DNA helicase, D3 proteins, flap endonuclease, ribonucleotide reductase, and DNA polymerase I, etc (Chim et al., 2018; Rozman Grinberg et al., 2018; Xu et al., 2018; Behrmann et al., 2021; Qureshi et al., 2021; Ren et al., 2021). Therefore, differences between the two phages in bacterial lysis, thermal stability and acid–base stability may be related to the proteins codified by gene 26 (NAD-dependent protein deacetylase of SIR2 family), gene 2 (flap endonuclease) and gene 28 (ribonucleotide reductase of class III anaerobic, large subunit) of phage CM_Kpn_HB132952, gene 50 (NAD-dependent protein deacetylase of SIR2 family), gene 30 (DNA helicase), gene 33 (DNA polymerase I), gene 43 (D3 protein), and gene 52 (ribonucleotide reductase of class III anaerobic, large subunit) of phage CM_Kpn_HB143742.

The genome diversity of bacteriophage is very high, and its origin is confusing. In this study, phages isolated from a sewage sample using drug-resistant and non-resistant *K*. *pneumoniae* strains were different in morphological structure, biological and genomic characteristics; therefore, contingency on the phage isolation by different host strains cannot be excluded. In addition, protective or therapeutic functions of phages isolated by strains with different antimicrobial resistance should be well characterized both *in vitro* and *in vivo*.

## Conclusion

The 2 bacteriophages were isolated from the same sewage sample using 1 MDR and 1 non-MDR *K*. *pneumoniae*. These bacteriophages differed in ultrastructure, MOI and 1-step growth curve, host range, and genomic characteristics, but had the same host range rate. This provided a reference basis for selection of strains during phage isolation by *K*. *pneumoniae* from bovine mastitis.

## Data availability statement

The datasets presented in this study can be found in online repositories. The names of the repository/repositories and accession number(s) can be found in the article/Supplementary material.

## Ethics statement

The animal study was reviewed and approved by Beijing Municipality Administration Office of Laboratory Animals (BAOLA); China Agricultural University Animal Ethics Committee (protocol CAU-AEC-2010–0603).

## Author contributions

JG and BH performed the research project administration. JG and BL conceived and designed the experiment. BL and WZ performed the research and wrote the original draft. HB, YN, and JK assisted in the data analyses, as well as revised and re-edited the manuscript. All authors read and approved the final manuscript.

## Funding

This study was financially supported by the Beijing-Tianjin-Hebei Collaborative Innovation Community Project (21346601D) and the National Natural Science Foundation of China (U21A20262 and 32172928).

## Conflict of interest

The authors declare that the research was conducted in the absence of any commercial or financial relationships that could be construed as a potential conflict of interest.

## Publisher’s note

All claims expressed in this article are solely those of the authors and do not necessarily represent those of their affiliated organizations, or those of the publisher, the editors and the reviewers. Any product that may be evaluated in this article, or claim that may be made by its manufacturer, is not guaranteed or endorsed by the publisher.

## Supplementary material

The Supplementary material for this article can be found online at: https://www.frontiersin.org/articles/10.3389/fmicb.2022.943279/full#supplementary-material

## Footnotes

^1^ http://www.usadellab.org/cms/uploads/supplementary/Trimmomatic

^2^ http://www.bcgsc.ca/platform/bioinfo/software/abyss

^3^ https://sourceforge.net/projects/soapdenovo2/files/GapCloser/

^4^ http://uniprot.org

^5^ http://www.genome.jp/kegg/

^6^ http://www.ncbi.nlm.nih.gov/COG

^7^ http://www.geneontology.org/
