# Supplementary material for: Biological and genomic characteristics of two bacteriophages isolated from sewage, using one multidrug-resistant and one non-multidrug-resistant strain of Klebsiella pneumoniae
Source: Front Microbiol. 2022 Oct 13;13:943279. doi: 10.3389/fmicb.2022.943279 (PMC9608510; doi:10.3389/fmicb.2022.943279)
Supplement: Supplementary file 1 [file Data_Sheet_1.docx]

**Supplementary Material**

**Supplementary Table 1 A**nnotation information for all predicted genes in NCBI between phage CM_Kpn_HB132952 and CM_Kpn_HB143742. Compared to phage CM_Kpn_HB143742, different predicted genes of phage CM_Kpn_HB132952 are in red. Compared to phage CM_Kpn_HB132952, different predicted genes of phage CM_Kpn_HB143742 are in blue.

| CM_Kpn_HB132952  Gene ID | CM_Kpn_HB143742  Gene ID | Length | NCBI Name | NCBI Description | Similar bases (%) |
| --- | --- | --- | --- | --- | --- |
| 15 | 38 | 320 | AOZ65432.1 | NAD-dependent DNA ligase subunit A [Klebsiella phage vB_Kpn_IME260] | 99.4 |
| 10 | 32 | 856/608 | AOZ65437.1 | DNA polymerase I [Klebsiella phage vB_Kpn_IME260] | 100 |
| 1 | 23 | 147 | AOZ65446.1 | deoxyuridine 5'-triphosphate nucleotidohydrolase [Klebsiella phage vB_Kpn_IME260] | 99.3 |
| 137 | 19 | 948 | AOZ65450.1 | tail length tape-measure protein [Klebsiella phage vB_Kpn_IME260] | 99.9 |
| 131 | 13 | 375 | AOZ65456.1 | tail fibers protein [Klebsiella phage vB_Kpn_IME260] | 100 |
| 31 | 56 | 794 | AOZ65459.2 | ribonucleotide reductase of class Ia (aerobic), alpha subunit [Klebsiella phage vB_Kpn_IME260] | 100 |
| 124 | 6 | 403 | AOZ65463.1 | portal (connector) protein [Klebsiella phage vB_Kpn_IME260] | 100 |
| 118 | 145 | 74 | AOZ65468.1 | hypothetical protein [Klebsiella phage vB_Kpn_IME260] | 100 |
| 117 | 144 | 240 | APT41049.1 | deoxynucleoside-5'-monophosphatase [Klebsiella phage vB_Kpn_IME260] | 98.3 |
| 116 | 143 | 150 | APT41050.1 | hypothetical protein [Klebsiella phage vB_Kpn_IME260] | 99.3 |
| 111 | 138 | 86 | APT41054.1 | hypothetical protein [Klebsiella phage vB_Kpn_IME260] | 97.7 |
| 80 | 106 | 134/126 | APT41081.1 | hypothetical protein [Klebsiella phage vB_Kpn_IME260] | 100 |
| 77 | 103 | 203 | APT41084.1 | ATP-dependent Clp protease proteolytic subunit [Klebsiella phage vB_Kpn_IME260] | 100 |
| 61 | 87 | 62 | APT41099.1 | hypothetical protein [Klebsiella phage vB_Kpn_IME260] | 98.3 |
| 37 | 62 | 160 | APT41122.1 | ribonuclease HI [Klebsiella phage vB_Kpn_IME260] | 100 |
| 33 | 58 | 187 | APT41126.1 | hypothetical protein [Klebsiella phage vB_Kpn_IME260] | 99.5 |
| 27 | 51 | 70 | APT41130.1 | hypothetical protein [Klebsiella phage vB_Kpn_IME260] | 100 |
| 128 | 10 | 167 | APT41142.1 | head completion protein [Klebsiella phage vB_Kpn_IME260] | 100 |
| 115 | 142 | 560 | APT41145.1 | putative A1 protein [Klebsiella phage vB_Kpn_IME260] | 99.5 |
| 105 | 132 | 321 | APT41154.1 | hypothetical protein [Klebsiella phage vB_Kpn_IME260] | 99.1 |
| 104 | 131 | 127 | APT41155.1 | S9 family peptidase [Klebsiella phage vB_Kpn_IME260] | 100 |
| 103 | 129 | 56 | APT41156.1 | nucleoside transporter [Klebsiella phage vB_Kpn_IME260] | 94.6 |
| 114 | 141 | 75 | ATW61841.1 | hypothetical protein CPT_Sugarland_004 [Klebsiella phage Sugarland] | 100 |
| 113 | 140 | 89 | ATW61842.1 | hypothetical protein CPT_Sugarland_005 [Klebsiella phage Sugarland] | 97.8 |
| 112 | 139 | 134 | ATW61843.1 | A2 protein [Klebsiella phage Sugarland] | 100 |
| 110 | 137 | 62 | ATW61845.1 | hypothetical protein CPT_Sugarland_008 [Klebsiella phage Sugarland] | 98.4 |
| 109 | 136 | 55 | ATW61846.1 | hypothetical protein CPT_Sugarland_009 [Klebsiella phage Sugarland] | 100 |
| 107 | 134 | 174 | ATW61849.1 | hypothetical protein CPT_Sugarland_012 [Klebsiella phage Sugarland] | 99.4 |
| 106 | 133 | 164 | ATW61850.1 | hypothetical protein CPT_Sugarland_013 [Klebsiella phage Sugarland] | 100 |
| 97 | 123 | 158 | ATW61860.1 | hypothetical protein CPT_Sugarland_023 [Klebsiella phage Sugarland] | 99.4 |
| 92 | 119 | 75 | ATW61865.1 | hypothetical protein CPT_Sugarland_028 [Klebsiella phage Sugarland] | 100 |
| 81 | 107 | 94 | ATW61877.1 | thioredoxin [Klebsiella phage Sugarland] | 100 |
| 76 | 102 | 253 | ATW61883.1 | deoxynucleotide 5'-monophosphate kinase [Klebsiella phage Sugarland] | 100 |
| 66 | 92 | 122 | ATW61895.1 | hypothetical protein CPT_Sugarland_058 [Klebsiella phage Sugarland] | 99.2 |
| 62 | 88 | 71 | ATW61899.1 | hypothetical protein CPT_Sugarland_062 [Klebsiella phage Sugarland] | 98.5 |
| 57 | 82 | 49 | ATW61905.1 | hypothetical protein CPT_Sugarland_072 [Klebsiella phage Sugarland] | 100 |
| 46 | 71 | 69 | ATW61925.1 | hypothetical protein CPT_Sugarland_111 [Klebsiella phage Sugarland] | 100 |
| 38 | 63 | 53 | ATW61938.1 | hypothetical protein CPT_Sugarland_125 [Klebsiella phage Sugarland] | 100 |
| 34 | 59 | 284 | ATW61942.1 | thymidylate synthase [Klebsiella phage Sugarland] | 99.6 |
| 133 | 15 | 140 | ATW61990.1 | putative tape measure chaperone [Klebsiella phage Sugarland] | 100 |
| 120 | 2 | 157 | ATW62003.1 | terminase small subunit [Klebsiella phage Sugarland] | 99.4 |
| 108 | 135 | 115 | QBX06873.1 | hypothetical protein CPT_Spivey_015 [Klebsiella phage Spivey] | 99.1 |
| 119 | 1 | 658 | QBX06877.1 | receptor-binding tail protein [Klebsiella phage Spivey] | 97.9 |
| 136 | 18 | 204 | QBX06894.1 | distal tail protein [Klebsiella phage Spivey] | 100 |
| 5 | 27 | 330 | QBX06906.1 | recombination endonuclease subunit [Klebsiella phage Spivey] | 99.1 |
| 11 | 34 | 213 | QBX06912.1 | putative DNA replication primase [Klebsiella phage Spivey] | 100 |
| 49 | 74 | 64 | QBX06960.1 | hypothetical protein CPT_Spivey_112 [Klebsiella phage Spivey] | 100 |
| 54 | 79 | 158 | QBX06969.1 | hypothetical protein CPT_Spivey_126 [Klebsiella phage Spivey] | 89.9 |
| 55 | 80 | 68 | QBX06970.1 | hypothetical protein CPT_Spivey_129 [Klebsiella phage Spivey] | 100 |
| 101 | 127 | 145 | QBX07023.1 | hypothetical protein CPT_Spivey_188 [Klebsiella phage Spivey] | 82.1 |
| 73 | 99 | 130 | QCG76528.1 | 6-phosphofructokinase [Klebsiella phage vB_KpnS_FZ41] | 93.1 |
| 4 | 26 | 598 | QCG76560.1 | putative exonuclease subunit 2 [Klebsiella phage vB_KpnS_FZ41] | 99.8 |
| 135 | 17 | 1485 | QEA03136.1 | tail tip protein [Klebsiella phage KpGranit] | 98.5 |
| 32 | 57 | 386 | QEA03152.1 | aerobic ribonucleoside diphosphate reductase, small subunit [Klebsiella phage KpGranit] | 99.2 |
| 132 | 14 | 290 | QEA03159.1 | minor tail protein [Klebsiella phage KpGranit] | 99 |
| 129 | 11 | 253 | QEA03167.1 | hypothetical protein [Klebsiella phage KpGranit] | 99.6 |
| 125 | 7 | 239 | QEA03172.1 | hypothetical protein [Klebsiella phage KpGranit] | 95.8 |
| 134 | 16 | 142 | QEA03210.1 | hypothetical protein [Klebsiella phage KpGranit] | 99.3 |
| 82 | 108 | 134 | QEA03217.1 | hypothetical protein [Klebsiella phage KpGranit] | 99.3 |
| 88 | 115 | 101 | QEA03244.1 | hypothetical protein [Klebsiella phage KpGranit] | 100 |
| 95 | 122 | 95 | QEA03246.1 | hypothetical protein [Klebsiella phage KpGranit] | 98.9 |
| 20 | 44 | 72 | QEG11226.1 | hypothetical protein KPN4_40 [Klebsiella phage KPN4] | 97.2 |
| 23 | 47 | 929 | QEG11229.1 | putative replication origin binding protein [Klebsiella phage KPN4] | 99.7 |
| 29 | 53 | 249 | QEG11237.1 | phosphate starvation-inducible protein [Klebsiella phage KPN4] | 100 |
| 43 | 68 | 108 | QEG11252.1 | hypothetical protein KPN4_66 [Klebsiella phage KPN4] | 98.1 |
| 67 | 93 | 45 | QEG11281.1 | hypothetical protein KPN4_95 [Klebsiella phage KPN4] | 100 |
| 69 | 95 | 134 | QEG11284.1 | hypothetical protein KPN4_98 [Klebsiella phage KPN4] | 99.3 |
| 72 | 98 | 147 | QEG11287.1 | hypothetical protein KPN4_101 [Klebsiella phage KPN4] | 100 |
| 74 | 100 | 237 | QEG11289.1 | hypothetical protein KPN4_103 [Klebsiella phage KPN4] | 99.2 |
| 79 | 105 | 137 | QEG11294.1 | endolysin [Klebsiella phage KPN4] | 100 |
| 87 | 114 | 142 | QEG11304.1 | hypothetical protein KPN4_118 [Klebsiella phage KPN4] | 100 |
| 90 | 117 | 82 | QEG11307.1 | hypothetical protein KPN4_121 [Klebsiella phage KPN4] | 98.8 |
| 91 | 118 | 113 | QEG11308.1 | hypothetical protein KPN4_122 [Klebsiella phage KPN4] | 100 |
| 94 | 121 | 123 | QEG11311.1 | hypothetical protein KPN4_125 [Klebsiella phage KPN4] | 99.2 |
| 102 | 128 | 245 | QEG11319.1 | hypothetical protein KPN4_133 [Klebsiella phage KPN4] | 99.6 |
| 99 | 125 | 169 | QFR57291.1 | hypothetical protein AmPhEK80_0021 [Klebsiella phage AmPh_EK80] | 98.2 |
| 98 | 124 | 133 | QFR57292.1 | hypothetical protein AmPhEK80_0022 [Klebsiella phage AmPh_EK80] | 97.7 |
| 89 | 116 | 93 | QFR57301.1 | hypothetical protein AmPhEK80_0031 [Klebsiella phage AmPh_EK80] | 100 |
| 75 | 101 | 153 | QFR57318.1 | hypothetical protein AmPhEK80_0048 [Klebsiella phage AmPh_EK80] | 98.7 |
| 64 | 90 | 176/180 | QFR57330.1 | hypothetical protein AmPhEK80_0060 [Klebsiella phage AmPh_EK80] | 100 |
| 60 | 86 | 126 | QFR57334.1 | hypothetical protein AmPhEK80_0064 [Klebsiella phage AmPh_EK80] | 100 |
| 59 | 85 | 118 | QFR57335.1 | hypothetical protein AmPhEK80_0065 [Klebsiella phage AmPh_EK80] | 99.2 |
| 42 | 67 | 153 | QFR57358.1 | hypothetical protein AmPhEK80_0108 [Klebsiella phage AmPh_EK80] | 100 |
| 41 | 66 | 334 | QFR57359.1 | hypothetical protein AmPhEK80_0109 [Klebsiella phage AmPh_EK80] | 99.4 |
| 40 | 65 | 136 | QFR57360.1 | hypothetical protein AmPhEK80_0110 [Klebsiella phage AmPh_EK80] | 100 |
| 39 | 64 | 116 | QFR57361.1 | hypothetical protein AmPhEK80_0111 [Klebsiella phage AmPh_EK80] | 96.6 |
| 36 | 61 | 93 | QFR57364.1 | hypothetical protein AmPhEK80_0114 [Klebsiella phage AmPh_EK80] | 100 |
| 21 | 45 | 231 | QFR57382.1 | hypothetical protein AmPhEK80_0132 [Klebsiella phage AmPh_EK80] | 100 |
| 18 | 42 | 98 | QFR57385.1 | hypothetical protein AmPhEK80_0135 [Klebsiella phage AmPh_EK80] | 100 |
| 16 | 39 | 91 | QFR57388.1 | hypothetical protein AmPhEK80_0138 [Klebsiella phage AmPh_EK80] | 83.5 |
| 127 | 9 | 460 | QFR57420.1 | major capsid protein [Klebsiella phage AmPh_EK80] | 99.8 |
| 126 | 8 | 198 | QFR57421.1 | capsid and scaffold protein [Klebsiella phage AmPh_EK80] | 100 |
| 121 | 3 | 438 | QFR57426.1 | terminase large subunit [Klebsiella phage AmPh_EK80] | 99.8 |
| 86 | 112 | 122 | QFR57465.1 | hypothetical protein JIPhKp127_0035 [Klebsiella phage JIPh_Kp127] | 99.2 |
| 85 | 111 | 77 | QFR57466.1 | hypothetical protein JIPhKp127_0036 [Klebsiella phage JIPh_Kp127] | 98.7 |
| 84 | 110 | 276 | QFR57467.1 | serine/threonine protein phosphatase [Klebsiella phage JIPh_Kp127] | 99.3 |
| 83 | 109 | 120 | QFR57468.1 | hypothetical protein JIPhKp127_0038 [Klebsiella phage JIPh_Kp127] | 100 |
| 70 | 96 | 107 | QFR57482.1 | hypothetical protein JIPhKp127_0052 [Klebsiella phage JIPh_Kp127] | 100 |
| 68 | 94 | 101 | QFR57485.1 | hypothetical protein JIPhKp127_0055 [Klebsiella phage JIPh_Kp127] | 100 |
| 65 | 91 | 75 | QFR57488.1 | hypothetical protein JIPhKp127_0058 [Klebsiella phage JIPh_Kp127] | 98.7 |
| 63 | 89 | 90 | QFR57490.1 | hypothetical protein JIPhKp127_0060 [Klebsiella phage JIPh_Kp127] | 93.3 |
| 58 | 84 | 63 | QFR57495.1 | hypothetical protein JIPhKp127_0065 [Klebsiella phage JIPh_Kp127] | 100 |
| 56 | 81 | 64 | QFR57498.1 | hypothetical protein JIPhKp127_0071 [Klebsiella phage JIPh_Kp127] | 100 |
| 53 | 78 | 71 | QFR57501.1 | hypothetical protein JIPhKp127_0079 [Klebsiella phage JIPh_Kp127] | 100 |
| 52 | 77 | 125 | QFR57502.1 | hypothetical protein JIPhKp127_0080 [Klebsiella phage JIPh_Kp127] | 100 |
| 51 | 76 | 90 | QFR57503.1 | hypothetical protein JIPhKp127_0081 [Klebsiella phage JIPh_Kp127] | 100 |
| 50 | 75 | 52 | QFR57504.1 | hypothetical protein JIPhKp127_0085 [Klebsiella phage JIPh_Kp127] | 100 |
| 47 | 72 | 164 | QFR57506.1 | hypothetical protein JIPhKp127_0095 [Klebsiella phage JIPh_Kp127] | 100 |
| 45 | 70 | 65 | QFR57508.1 | hypothetical protein JIPhKp127_0097 [Klebsiella phage JIPh_Kp127] | 100 |
| 44 | 69 | 92 | QFR57509.1 | hypothetical protein JIPhKp127_0099 [Klebsiella phage JIPh_Kp127] | 98.9 |
| 35 | 60 | 77 | QFR57520.1 | hypothetical protein JIPhKp127_0112 [Klebsiella phage JIPh_Kp127] | 100 |
| 30 | 55 | 69 | QFR57526.1 | hypothetical protein JIPhKp127_0118 [Klebsiella phage JIPh_Kp127] | 100 |
| 25 | 49 | 133 | QFR57533.1 | hypothetical protein JIPhKp127_0125 [Klebsiella phage JIPh_Kp127] | 99.2 |
| 22 | 46 | 94 | QFR57536.1 | hypothetical protein JIPhKp127_0128 [Klebsiella phage JIPh_Kp127] | 100 |
| 17 | 41 | 102 | QFR57541.1 | hypothetical protein JIPhKp127_0133 [Klebsiella phage JIPh_Kp127] | 100 |
| 14 | 37 | 249 | QFR57544.1 | DNA ligase [Klebsiella phage JIPh_Kp127] | 99.6 |
| 13 | 36 | 252 | QFR57545.1 | hypothetical protein JIPhKp127_0137 [Klebsiella phage JIPh_Kp127] | 100 |
| 12 | 35 | 492 | QFR57546.1 | recombinase [Klebsiella phage JIPh_Kp127] | 100 |
| 9 | 31 | 165 | QFR57549.1 | hypothetical protein JIPhKp127_0141 [Klebsiella phage JIPh_Kp127] | 100 |
| 7 | 29 | 117 | QFR57551.1 | hypothetical protein JIPhKp127_0143 [Klebsiella phage JIPh_Kp127] | 100 |
| 6 | 28 | 257 | QFR57552.1 | hypothetical protein JIPhKp127_0144 [Klebsiella phage JIPh_Kp127] | 100 |
| 3 | 25 | 160 | QFR57555.1 | hypothetical protein JIPhKp127_0147 [Klebsiella phage JIPh_Kp127] | 100 |
| 130 | 12 | 161 | QFR57568.1 | hypothetical protein JIPhKp127_0160 [Klebsiella phage JIPh_Kp127] | 98.8 |
| 123 | 5 | 146 | QFR57575.1 | hypothetical protein JIPhKp127_0167 [Klebsiella phage JIPh_Kp127] | 99.3 |
| 8 | 30 | 452 | QFR57550.1 | DNA helicase [Klebsiella phage JIPh_Kp127] | 99.8 |
| 93 | 30 | 155/452 | QFR57550.1 | DNA helicase [Klebsiella phage JIPh_Kp127] | 99.8 |
| 24 | - | 131 | QFR57534.1 | hypothetical protein JIPhKp127_0126 [Klebsiella phage JIPh_Kp127] | 100 |
| 96 | - | 62 | QFR57454.1 | hypothetical protein JIPhKp127_0024 [Klebsiella phage JIPh_Kp127] | 100 |
| 19 | - | 134 | QFR57384.1 | hypothetical protein AmPhEK80_0134 [Klebsiella phage AmPh_EK80] | 100 |
| 2 | - | 292 | QEG11209.1 | flap endonuclease [Klebsiella phage KPN4] | 100 |
| 100 | - | 104 | QEG11317.1 | hypothetical protein KPN4_131 [Klebsiella phage KPN4] | 98.1 |
| 71 | - | 90 | QBX06989.1 | hypothetical protein CPT_Spivey_154 [Klebsiella phage Spivey] | 100 |
| 78 | - | 220 | QBX06997.1 | putative holin [Klebsiella phage Spivey] | 100 |
| 140 | - | 680 | QEA03141.1 | putative tail fiber protein [Klebsiella phage KpGranit] | 100 |
| 139 | - | 139 | QEA03212.1 | putative tail protein [Klebsiella phage KpGranit] | 100 |
| 138 | - | 3209 | QEA03135.1 | tail fiber protein [Klebsiella phage KpGranit] | 96.9 |
| 28 | - | 609 | AOZ65464.2 | ribonucleotide reductase of class III (anaerobic), large subunit [Klebsiella phage vB_Kpn_IME260] | 99.2 |
| 26 | - | 277 | APT41131.1 | NAD-dependent protein deacetylase of SIR2 family [Klebsiella phage vB_Kpn_IME260] | 99.6 |
| - | 24 | 292 | QFR57556.1 | ribonuclease H [Klebsiella phage JIPh_Kp127] | 100 |
| - | 52 | 609 | QFR57529.1 | ribonucleotide reductase of class III (anaerobic), large subunit [Klebsiella phage JIPh_Kp127] | 99.5 |
| - | 83 | 33 | QFR57496.1 | hypothetical protein JIPhKp127_0066 [Klebsiella phage JIPh_Kp127] | 100 |
| - | 120 | 155 | QFR57457.1 | hypothetical protein JIPhKp127_0027 [Klebsiella phage JIPh_Kp127] | 100 |
| - | 20 | 3477 | AOZ65449.1 | tail fiber protein [Klebsiella phage vB_Kpn_IME260] | 99.5 |
| - | 21 | 139 | AOZ65448.1 | tail protein [Klebsiella phage vB_Kpn_IME260] | 100 |
| - | 40 | 45 | AOZ65430.1 | hypothetical protein [Klebsiella phage vB_Kpn_IME260] | 100 |
| - | 22 | 679 | QEA03141.1 | putative tail fiber protein [Klebsiella phage KpGranit] | 98.2 |
| - | 48 | 131 | QEA03223.1 | hypothetical protein [Klebsiella phage KpGranit] | 100 |
| - | 33 | 241 | QEG11216.1 | DNA polymerase I [Klebsiella phage KPN4] | 99.6 |
| - | 104 | 220 | QEG11293.1 | holin [Klebsiella phage KPN4] | 99.5 |
| - | 126 | 104 | QEG11317.1 | hypothetical protein KPN4_131 [Klebsiella phage KPN4] | 98.1 |
| - | 43 | 134 | QCG76548.1 | D3 protein [Klebsiella phage vB_KpnS_FZ41] | 99.3 |
| - | 50 | 277 | QFR57376.1 | NAD-dependent protein deacetylase of SIR2 family [Klebsiella phage AmPh_EK80] | 99.3 |
| - | 54 | 37 | QFR57372.1 | hypothetical protein AmPhEK80_0122 [Klebsiella phage AmPh_EK80] | 100 |
| - | 97 | 90 | QFR57322.1 | hypothetical protein AmPhEK80_0052 [Klebsiella phage AmPh_EK80] | 100 |
| - | 113 | 39 | QFR57304.1 | hypothetical protein AmPhEK80_0034 [Klebsiella phage AmPh_EK80] | 100 |
| - | 130 | 46 | ATW61853.1 | hypothetical protein CPT_Sugarland_016 [Klebsiella phage Sugarland] | 89.1 |

**Supplementary Table 2 A**nnotation information for all predicted genes in GO between phage CM_Kpn_HB132952 and CM_Kpn_HB143742. Compared to phage CM_Kpn_HB143742, different predicted genes of phage CM_Kpn_HB132952 are in red. Compared to phage CM_Kpn_HB132952, different predicted genes of phage CM_Kpn_HB143742 are in blue.

| CM_Kpn_HB132952  Gene ID | CM_Kpn_HB143742  Gene ID | Length | GO ID |
| --- | --- | --- | --- |
| 10 | 32 | 856/608 | GO:0003674;GO:0003824;GO:0003887;GO:0004518;GO:0004527;GO:0006139;GO:0006259;GO:0006725;GO:0006807;GO:0008150;GO:0008152;GO:0008408;GO:0009058;GO:0009059;GO:0009987;GO:0016740;GO:0016772;GO:0016779;GO:0016787;GO:0016788;GO:0018130;GO:0019438;GO:0034061;GO:0034641;GO:0034645;GO:0034654;GO:0043170;GO:0044237;GO:0044238;GO:0044249;GO:0044260;GO:0044271;GO:0046483;GO:0071704;GO:0071897;GO:0090304;GO:0090305;GO:0140097;GO:1901360;GO:1901362;GO:1901576 |
| 1 | 23 | 147 | GO:0003674;GO:0003824;GO:0004170;GO:0016462;GO:0016787;GO:0016817;GO:0016818;GO:0047429 |
| 137 | 19 | 948 | GO:0005575;GO:0008150;GO:0016032;GO:0018995;GO:0019012;GO:0019058;GO:0019062;GO:0022610;GO:0030430;GO:0033643;GO:0033646;GO:0043656;GO:0043657;GO:0044215;GO:0044216;GO:0044217;GO:0044403;GO:0044406;GO:0044419;GO:0044650;GO:0051704 |
| 131 | 13 | 375 | GO:0005575;GO:0019012;GO:0019028;GO:0019030;GO:0039617;GO:0044423;GO:0046729;GO:0098015 |
| 31 | 56 | 794 | GO:0001959;GO:0001960;GO:0003674;GO:0003824;GO:0004748;GO:0005575;GO:0008150;GO:0008152;GO:0009966;GO:0009968;GO:0010646;GO:0010648;GO:0010803;GO:0010804;GO:0016491;GO:0016725;GO:0016728;GO:0018995;GO:0023051;GO:0023057;GO:0030430;GO:0033643;GO:0033646;GO:0043656;GO:0043657;GO:0044215;GO:0044216;GO:0044217;GO:0048519;GO:0048523;GO:0048583;GO:0048585;GO:0050789;GO:0050794;GO:0055114;GO:0060759;GO:0060761;GO:0061731;GO:0065007 |
| 117 | 144 | 240 | GO:0008150;GO:0016032;GO:0019048;GO:0035821;GO:0039637;GO:0044003;GO:0044403;GO:0044419;GO:0051701;GO:0051704;GO:0051817 |
| 128 | 10 | 167 | GO:0008150;GO:0016032;GO:0019058;GO:0019068;GO:0019069;GO:0044403;GO:0044419;GO:0051704 |
| 76 | 102 | 253 | GO:0003674;GO:0003824;GO:0006793;GO:0006796;GO:0008150;GO:0008152;GO:0009987;GO:0016301;GO:0016310;GO:0016740;GO:0016772;GO:0016776;GO:0019205;GO:0044237;GO:0047507 |
| 34 | 59 | 284 | GO:0003674;GO:0003824;GO:0004799;GO:0008150;GO:0008152;GO:0008168;GO:0016740;GO:0016741;GO:0032259;GO:0042083 |
| 119 | 1 | 658 | GO:0008150;GO:0016032;GO:0019058;GO:0019062;GO:0022610;GO:0044403;GO:0044406;GO:0044419;GO:0044650;GO:0046813;GO:0051704 |
| 136 | 18 | 204 | GO:0005575;GO:0019012;GO:0044423;GO:0098015 |
| 135 | 17 | 1485 | GO:0005575;GO:0008150;GO:0016032;GO:0019012;GO:0019058;GO:0019068;GO:0044403;GO:0044409;GO:0044411;GO:0044419;GO:0044423;GO:0051701;GO:0051704;GO:0051828;GO:0051830;GO:0085027;GO:0098003;GO:0098015 |
| 132 | 14 | 290 | GO:0005575;GO:0019012;GO:0044423;GO:0098015 |
| 129 | 11 | 253 | GO:0005575;GO:0008150;GO:0016032;GO:0019012;GO:0019058;GO:0019068;GO:0044403;GO:0044419;GO:0044423;GO:0051704;GO:0098003;GO:0098015 |
| 23 | 47 | 929 | GO:0003674;GO:0003676;GO:0003677;GO:0003688;GO:0003690;GO:0003824;GO:0004386;GO:0005488;GO:0005575;GO:0008150;GO:0016032;GO:0016462;GO:0016787;GO:0016817;GO:0016818;GO:0017111;GO:0018995;GO:0019058;GO:0019079;GO:0033643;GO:0033646;GO:0033647;GO:0033648;GO:0039686;GO:0039693;GO:0042025;GO:0043565;GO:0043656;GO:0043657;GO:0044215;GO:0044216;GO:0044217;GO:0044403;GO:0044419;GO:0051704;GO:0097159;GO:1901363;GO:1990837 |
| 79 | 105 | 137 | GO:0001906;GO:0001907;GO:0003674;GO:0003824;GO:0006508;GO:0006807;GO:0008150;GO:0008152;GO:0008233;GO:0016032;GO:0016787;GO:0019048;GO:0019058;GO:0019076;GO:0019538;GO:0031640;GO:0035821;GO:0035890;GO:0035891;GO:0039633;GO:0040011;GO:0043170;GO:0044003;GO:0044004;GO:0044238;GO:0044364;GO:0044403;GO:0044419;GO:0044659;GO:0044661;GO:0051701;GO:0051704;GO:0051817;GO:0051818;GO:0051883;GO:0052126;GO:0052192;GO:0071704;GO:0140096;GO:1901564 |
| 127 | 9 | 460 | GO:0003674;GO:0003824;GO:0004553;GO:0005575;GO:0008150;GO:0009605;GO:0009607;GO:0016032;GO:0016787;GO:0016798;GO:0019012;GO:0019028;GO:0019048;GO:0019049;GO:0019058;GO:0019062;GO:0022610;GO:0032991;GO:0035821;GO:0043207;GO:0044003;GO:0044403;GO:0044406;GO:0044409;GO:0044411;GO:0044413;GO:0044415;GO:0044419;GO:0044423;GO:0044650;GO:0046806;GO:0050896;GO:0051701;GO:0051704;GO:0051707;GO:0051817;GO:0051828;GO:0051830;GO:0051832;GO:0051834;GO:0052173;GO:0052200;GO:0075136;GO:0085027;GO:0098015;GO:0098024;GO:0099018 |
| 126 | 8 | 198 | GO:0003674;GO:0003824;GO:0005575;GO:0006508;GO:0006807;GO:0008150;GO:0008152;GO:0008233;GO:0016787;GO:0019012;GO:0019538;GO:0043170;GO:0044238;GO:0071704;GO:0140096;GO:1901564 |
| 121 | 3 | 438 | GO:0003674;GO:0003824;GO:0004518;GO:0006139;GO:0006725;GO:0006807;GO:0008150;GO:0008152;GO:0009987;GO:0016787;GO:0016788;GO:0034641;GO:0043170;GO:0044237;GO:0044238;GO:0046483;GO:0071704;GO:0090304;GO:0090305;GO:1901360 |
| 130 | 12 | 161 | GO:0005575;GO:0019012;GO:0044423;GO:0098015 |
| 123 | 5 | 146 | GO:0003674;GO:0003824;GO:0004518;GO:0004519;GO:0006139;GO:0006725;GO:0006807;GO:0008150;GO:0008152;GO:0009987;GO:0016787;GO:0016788;GO:0034641;GO:0043170;GO:0044237;GO:0044238;GO:0046483;GO:0071704;GO:0090304;GO:0090305;GO:1901360 |
| 2 | - | 292 | GO:0003674;GO:0003824;GO:0004518;GO:0004519;GO:0004520;GO:0004521;GO:0004523;GO:0004527;GO:0004536;GO:0004540;GO:0006139;GO:0006259;GO:0006725;GO:0006807;GO:0008150;GO:0008152;GO:0008409;GO:0009987;GO:0016070;GO:0016787;GO:0016788;GO:0016888;GO:0016891;GO:0016893;GO:0017108;GO:0034641;GO:0043170;GO:0044237;GO:0044238;GO:0044260;GO:0046483;GO:0048256;GO:0071704;GO:0090304;GO:0090305;GO:0090501;GO:0090502;GO:0140097;GO:0140098;GO:1901360 |
| 78 | - | 220 | GO:0001906;GO:0001907;GO:0003674;GO:0005215;GO:0005575;GO:0006810;GO:0008150;GO:0015267;GO:0016032;GO:0018995;GO:0019048;GO:0019058;GO:0019076;GO:0020002;GO:0022803;GO:0022857;GO:0031640;GO:0033643;GO:0033644;GO:0034290;GO:0034291;GO:0035821;GO:0035890;GO:0035891;GO:0039633;GO:0040011;GO:0043657;GO:0044003;GO:0044004;GO:0044215;GO:0044216;GO:0044217;GO:0044218;GO:0044279;GO:0044364;GO:0044403;GO:0044419;GO:0044659;GO:0044660;GO:0044661;GO:0051179;GO:0051234;GO:0051701;GO:0051704;GO:0051817;GO:0051818;GO:0051883;GO:0052126;GO:0052192;GO:0055085 |
| 139 | - | 139 | GO:0005575;GO:0019012;GO:0044423;GO:0098015;GO:0098024 |
| 138 | - | 3209 | GO:0005575;GO:0019012;GO:0044423;GO:0098015 |
| - | 24 | 292 | GO:0003674;GO:0003824;GO:0004518;GO:0004519;GO:0004520;GO:0004521;GO:0004523;GO:0004527;GO:0004536;GO:0004540;GO:0006139;GO:0006259;GO:0006725;GO:0006807;GO:0008150;GO:0008152;GO:0008409;GO:0009987;GO:0016070;GO:0016787;GO:0016788;GO:0016888;GO:0016891;GO:0016893;GO:0017108;GO:0034641;GO:0043170;GO:0044237;GO:0044238;GO:0044260;GO:0046483;GO:0048256;GO:0071704;GO:0090304;GO:0090305;GO:0090501;GO:0090502;GO:0140097;GO:0140098;GO:1901360 |
| - | 20 | 3477 | GO:0005575;GO:0019012;GO:0044423;GO:0098015 |
| - | 21 | 139 | GO:0005575;GO:0019012;GO:0044423;GO:0098015;GO:0098024 |
| - | 33 | 241 | GO:0003674;GO:0003824;GO:0003887;GO:0004518;GO:0004527;GO:0006139;GO:0006259;GO:0006725;GO:0006807;GO:0008150;GO:0008152;GO:0008408;GO:0009058;GO:0009059;GO:0009987;GO:0016740;GO:0016772;GO:0016779;GO:0016787;GO:0016788;GO:0018130;GO:0019438;GO:0034061;GO:0034641;GO:0034645;GO:0034654;GO:0043170;GO:0044237;GO:0044238;GO:0044249;GO:0044260;GO:0044271;GO:0046483;GO:0071704;GO:0071897;GO:0090304;GO:0090305;GO:0140097;GO:1901360;GO:1901362;GO:1901576 |
| - | 104 | 220 | GO:0001906;GO:0001907;GO:0003674;GO:0005215;GO:0005575;GO:0006810;GO:0008150;GO:0015267;GO:0016032;GO:0018995;GO:0019048;GO:0019058;GO:0019076;GO:0020002;GO:0022803;GO:0022857;GO:0031640;GO:0033643;GO:0033644;GO:0034290;GO:0034291;GO:0035821;GO:0035890;GO:0035891;GO:0039633;GO:0040011;GO:0043657;GO:0044003;GO:0044004;GO:0044215;GO:0044216;GO:0044217;GO:0044218;GO:0044279;GO:0044364;GO:0044403;GO:0044419;GO:0044659;GO:0044660;GO:0044661;GO:0051179;GO:0051234;GO:0051701;GO:0051704;GO:0051817;GO:0051818;GO:0051883;GO:0052126;GO:0052192;GO:0055085 |

**Supplementary Table 3** Annotation information for all predicted genes in COG between phage CM_Kpn_HB132952 and CM_Kpn_HB143742.

| CM_Kpn_HB132952  Gene ID | CM_Kpn_HB143742  Gene ID | Length | COG ID | COG Function | COG Category | COG Description |
| --- | --- | --- | --- | --- | --- | --- |
| 125 | 7 | 239 | COG4412\|COG5492 | N | CELLULAR PROCESSES AND SIGNALING | Leucine-rich repeat (LRR) protein |

**Supplementary Table 4** Annotation information for all predicted genes in KEGG between phage CM_Kpn_HB132952 and CM_Kpn_HB143742. Compared to phage CM_Kpn_HB143742, different predicted genes of phage CM_Kpn_HB132952 are in red. Compared to phage CM_Kpn_HB132952, different predicted genes of phage CM_Kpn_HB143742 are in blue.

| CM_Kpn_HB132952  Gene ID | CM_Kpn_HB143742  Gene ID | Length | KEGG ID | Pathway | KEGG description |
| --- | --- | --- | --- | --- | --- |
| 15 | 38 | 320 | K01972 | ko03030, ko03410, ko03420, ko03430 | E6.5.1.2, ligA, ligB; DNA ligase (NAD+) [EC:6.5.1.2] |
| 1 | 23 | 147 | K01520 | ko00240, ko00983, ko01100 | dut, DUT; dUTP pyrophosphatase [EC:3.6.1.23] |
| 37 | 62 | 160 | K03469 | ko03030 | rnhA, RNASEH1; ribonuclease HI [EC:3.1.26.4] |
| 34 | 59 | 284 | K00560 | ko00240, ko00670, 01100, ko01523 | thyA, TYMS; thymidylate synthase [EC:2.1.1.45] |
| 11 | 34 | 213 | K02316 | ko03030 | dnaG; DNA primase [EC:2.7.7.101] |
| 32 | 57 | 386 | K00526 | ko00230, ko00240, ko01100 | E1.17.4.1B, nrdB, nrdF; ribonucleoside-diphosphate reductase beta chain [EC:1.17.4.1] |
| 42 | 67 | 153 | K01449 | - | cwlJ, sleB |
| 126 | 8 | 198 | K06904 | - | K06904 |
| 121 | 3 | 438 | K06909 | - | xtmB |
| 14 | 37 | 249 | K01972 | ko03030, ko03410, ko03420, ko03430 | E6.5.1.2, ligA, ligB; DNA ligase (NAD+) [EC:6.5.1.2] |
| 2 | - | 292 | K18950 | - | rnh |
| 28 | - | 609 | K21636 | ko00230, ko00240, ko01100 | nrdD; ribonucleoside-triphosphate reductase (formate) [EC:1.1.98.6] |
| 10 | - | 856 | K21314 | - | pol |
| - | 24 | 292 | K18950 | - | rnh |
| - | 52 | 609 | K21636 | ko00230, ko00240, ko01100 | nrdD; ribonucleoside-triphosphate reductase (formate) [EC:1.1.98.6] |

**Supplementary Table 5** Annotation information for all predicted genes in Swiss-port between phage CM_Kpn_HB132952 and CM_Kpn_HB143742. Compared to phage CM_Kpn_HB143742, different predicted genes of phage CM_Kpn_HB132952 are in red. Compared to phage CM_Kpn_HB132952, different predicted genes of phage CM_Kpn_HB143742 are in blue.

| CM_Kpn_HB132952  Gene ID | CM_Kpn_HB143742  Gene ID | Length | Swiss-port Name | Swiss-port description | Similar bases (%) |
| --- | --- | --- | --- | --- | --- |
| 15 | 38 | 320 | sp\|Q7VF74\|DNLJ_HELHP | DNA ligase OS=Helicobacter hepaticus (strain ATCC 51449 / 3B1) OX=235279 GN=ligA PE=3 SV=1 | 31.4 |
| 10 | 32 | 856/608 | sp\|P19822\|DPOL_BPT5 | DNA polymerase OS=Escherichia phage T5 OX=10726 GN=T5.122 PE=1 SV=3 | 77.5 |
| 1 | 23 | 147 | sp\|O48500\|DUT_BPT5 | Deoxyuridine 5'-triphosphate nucleotidohydrolase OS=Escherichia phage T5 OX=10726 GN=DUT PE=1 SV=1 | 71 |
| 137 | 19 | 948 | sp\|Q6QGE9\|BPPB3_BPT5 | Probable baseplate hub protein OS=Escherichia phage T5 OX=10726 GN=D16 PE=2 SV=1 | 72.6 |
| 131 | 13 | 375 | sp\|Q6QGE2\|TUBE_BPT5 | Tail tube protein OS=Escherichia phage T5 OX=10726 GN=N4 PE=1 SV=1 | 74.2 |
| 31 | 56 | 794 | sp\|P43754\|RIR1_HAEIN | Ribonucleoside-diphosphate reductase subunit alpha OS=Haemophilus influenzae (strain ATCC 51907 / DSM 11121 / KW20 / Rd) OX=71421 GN=nrdA PE=3 SV=1 | 34.3 |
| 124 | 6 | 403 | sp\|Q6QGD5\|PORTL_BPT5 | Portal protein OS=Escherichia phage T5 OX=10726 GN=ORF141 PE=1 SV=1 | 78.1 |
| 117 | 144 | 240 | sp\|Q38167\|DMP_BPT5 | 5'-deoxynucleotidase OS=Escherichia phage T5 OX=10726 GN=dmp PE=1 SV=2 | 60.9 |
| 111 | 138 | 86 | sp\|P19195\|Y9KD_BPBF2 | Uncharacterized 9.2 kDa protein OS=Escherichia phage Bf23 OX=10707 PE=4 SV=1 | 57 |
| 37 | 62 | 160 | sp\|Q0C3M1\|RNH_HYPNA | Ribonuclease H OS=Hyphomonas neptunium (strain ATCC 15444) OX=228405 GN=rnhA PE=3 SV=1 | 40.9 |
| 128 | 10 | 167 | sp\|Q6QGD9\|HCP_BPT5 | Head completion protein OS=Escherichia phage T5 OX=10726 GN=T5.148 PE=4 SV=1 | 67.7 |
| 115 | 142 | 560 | sp\|Q6QGT3\|A1_BPT5 | Protein A1 OS=Escherichia phage T5 OX=10726 GN=A1 PE=1 SV=1 | 73.8 |
| 112 | 139 | 134 | sp\|P23541\|A2_BPT5 | Protein A2 OS=Escherichia phage T5 OX=10726 GN=A2 PE=1 SV=2 | 66.2 |
| 76 | 102 | 253 | sp\|Q6QGP4\|DNMK_BPT5 | Deoxynucleoside-5'-monophosphate kinase OS=Escherichia phage T5 OX=10726 GN=dnk PE=1 SV=1 | 46.4 |
| 34 | 59 | 284 | sp\|Q5R064\|TYSY_IDILO | Thymidylate synthase OS=Idiomarina loihiensis (strain ATCC BAA-735 / DSM 15497 / L2-TR) OX=283942 GN=thyA PE=3 SV=1 | 65.9 |
| 120 | 2 | 157 | sp\|P23208\|TERS_BPT5 | Probable terminase, small subunit OS=Escherichia phage T5 OX=10726 GN=T5.156 PE=2 SV=2 | 61.6 |
| 119 | 1 | 658 | sp\|P23207\|RBP5_BPT5 | Receptor-binding protein OS=Escherichia phage T5 OX=10726 GN=oad PE=1 SV=2 | 40.1 |
| 136 | 18 | 204 | sp\|Q6QGE8\|DIT_BPT5 | Distal tail protein OS=Escherichia phage T5 OX=10726 GN=D16 PE=1 SV=1 | 63.7 |
| 5 | 27 | 330 | sp\|P11108\|EXO1_BPT5 | Probable exonuclease subunit 1 OS=Escherichia phage T5 OX=10726 GN=D12 PE=2 SV=2 | 65.5 |
| 11 | 34 | 213 | sp\|P33655\|DNAG_CLOAB | DNA primase OS=Clostridium acetobutylicum (strain ATCC 824 / DSM 792 / JCM 1419 / LMG 5710 / VKM B-1787) OX=272562 GN=dnaG PE=3 SV=1 | 30.3 |
| 4 | 26 | 598 | sp\|P11109\|EXO2_BPT5 | Probable exonuclease subunit 2 OS=Escherichia phage T5 OX=10726 GN=D13 PE=2 SV=1 | 62.5 |
| 135 | 17 | 1485 | sp\|Q6QGE7\|TMP_BPT5 | Probable tape measure protein OS=Escherichia phage T5 OX=10726 GN=D18-19 PE=1 SV=1 | 51.4 |
| 32 | 57 | 386 | sp\|P69924\|RIR2_ECOLI | Ribonucleoside-diphosphate reductase 1 subunit beta OS=Escherichia coli (strain K12) OX=83333 GN=nrdB PE=1 SV=2 | 35.4 |
| 132 | 14 | 290 | sp\|Q6QGE3\|TAIL1_BPT5 | Minor tail protein OS=Escherichia phage T5 OX=10726 GN=ORF133 PE=2 SV=1 | 56.7 |
| 129 | 11 | 253 | sp\|Q6QGE0\|COMPL_BPT5 | Tail completion protein OS=Escherichia phage T5 OX=10726 GN=ORF136 PE=2 SV=1 | 72.3 |
| 125 | 7 | 239 | sp\|P85991\|IGLVP_BPSK9 | Ig-like virion protein OS=Serratia phage KSP90 OX=552528 PE=1 SV=2 | 40.6 |
| 23 | 47 | 929 | sp\|Q6QGH9\|OBP_BPT5 | Putative replication origin binding protein OS=Escherichia phage T5 OX=10726 GN=obp PE=1 SV=1 | 78.9 |
| 29 | 53 | 249 | sp\|P0A9K6\|PHOL_SHIFL | PhoH-like protein OS=Shigella flexneri OX=623 GN=ybeZ PE=3 SV=2 | 37.6 |
| 79 | 105 | 137 | sp\|Q6QGP7\|ENLYS_BPT5 | L-alanyl-D-glutamate peptidase OS=Escherichia phage T5 OX=10726 GN=lys PE=1 SV=1 | 75.9 |
| 127 | 9 | 460 | sp\|Q6QGD8\|CAPSD_BPT5 | Major capsid protein OS=Escherichia phage T5 OX=10726 GN=D20 PE=1 SV=1 | 66.7 |
| 126 | 8 | 198 | sp\|Q6QGD7\|PRO_BPT5 | Prohead protease OS=Escherichia phage T5 OX=10726 GN=T5.150 PE=1 SV=1 | 59.7 |
| 121 | 3 | 438 | sp\|Q6QGD2\|TERL_BPT5 | Terminase, large subunit OS=Escherichia phage T5 OX=10726 GN=ORF144 PE=1 SV=1 | 88.8 |
| 84 | 110 | 276 | sp\|P03772\|PP_LAMBD | Serine/threonine-protein phosphatase OS=Escherichia phage lambda OX=10710 PE=1 SV=1 | 27.5 |
| 14 | 37 | 249 | sp\|Q5FLL4\|DNLJ_LACAC | DNA ligase OS=Lactobacillus acidophilus (strain ATCC 700396 / NCK56 / N2 / NCFM) OX=272621 GN=ligA PE=3 SV=1 | 29.8 |
| 13 | 36 | 252 | sp\|Q6QGG9\|D5_BPT5 | Putative transcription factor D5 OS=Escherichia phage T5 OX=10726 GN=D5 PE=1 SV=1 | 77.4 |
| 6 | 28 | 257 | sp\|P20376\|SSDNA_BPT5 | Probable ssDNA-binding protein OS=Escherichia phage T5 OX=10726 GN=D11 PE=2 SV=1 | 63.1 |
| 3 | 25 | 160 | sp\|O48499\|D14_BPT5 | Protein D14 OS=Escherichia phage T5 OX=10726 GN=D14 PE=2 SV=1 | 73.8 |
| 130 | 12 | 161 | sp\|Q6QGE1\|TTTP_BPT5 | Tail tube terminator protein OS=Escherichia phage T5 OX=10726 GN=ORF135 PE=2 SV=1 | 69.6 |
| 123 | 5 | 146 | sp\|Q6QGD4\|ENDON_BPT5 | Nicking endonuclease OS=Escherichia phage T5 OX=10726 GN=ORF142 PE=2 SV=1 | 63.4 |
| 8 | 30 | 452 | sp\|P11107\|HEL10_BPT5 | Probable helicase D10 OS=Escherichia phage T5 OX=10726 GN=D10 PE=2 SV=1 | 68 |
| 93 | 30 | 155/452 | sp\|P11107\|HEL10_BPT5 | Probable helicase D10 OS=Escherichia phage T5 OX=10726 GN=D10 PE=2 SV=1 | 68 |
| 2 | - | 292 | sp\|P06229\|FEN_BPT5 | Flap endonuclease OS=Escherichia phage T5 OX=10726 GN=D15 PE=1 SV=3 | 70.2 |
| 78 | - | 220 | sp\|Q6R6U4\|HOLIN_BPT5 | Holin OS=Escherichia phage T5 OX=10726 GN=C1 PE=2 SV=1 | 65.5 |
| 139 | - | 139 | sp\|Q7Y5D9\|FIBL2_BPT5 | L-shaped tail fiber protein p132 OS=Escherichia phage T5 OX=10726 GN=ORF125 PE=2 SV=1 | 58.3 |
| 138 | - | 3209 | sp\|Q6QGF0\|FIBC_BPT5 | Probable central straight fiber OS=Escherichia phage T5 OX=10726 GN=D17 PE=2 SV=1 | 60 |
| 28 | - | 609 | sp\|Q9L646\|NRDD_SALTY | Anaerobic ribonucleoside-triphosphate reductase OS=Salmonella typhimurium (strain LT2 / SGSC1412 / ATCC 700720) OX=99287 GN=nrdD PE=3 SV=1 | 53.5 |
| 26 | - | 277 | sp\|Q7VIN2\|NPD_HELHP | NAD-dependent protein deacylase OS=Helicobacter hepaticus (strain ATCC 51449 / 3B1) OX=235279 GN=cobB PE=3 SV=1 | 36 |
| - | 24 | 292 | sp\|P06229\|FEN_BPT5 | Flap endonuclease OS=Escherichia phage T5 OX=10726 GN=D15 PE=1 SV=3 | 70.2 |
| - | 52 | 609 | sp\|Q9L646\|NRDD_SALTY | Anaerobic ribonucleoside-triphosphate reductase OS=Salmonella typhimurium (strain LT2 / SGSC1412 / ATCC 700720) OX=99287 GN=nrdD PE=3 SV=1 | 53.4 |
| - | 20 | 3477 | sp\|Q6QGF0\|FIBC_BPT5 | Probable central straight fiber OS=Escherichia phage T5 OX=10726 GN=D17 PE=2 SV=1 | 60 |
| - | 21 | 139 | sp\|Q7Y5D9\|FIBL2_BPT5 | L-shaped tail fiber protein p132 OS=Escherichia phage T5 OX=10726 GN=ORF125 PE=2 SV=1 | 59.7 |
| - | 33 | 241 | sp\|P19822\|DPOL_BPT5 | DNA polymerase OS=Escherichia phage T5 OX=10726 GN=T5.122 PE=1 SV=3 | 75.7 |
| - | 104 | 220 | sp\|Q6R6U4\|HOLIN_BPT5 | Holin OS=Escherichia phage T5 OX=10726 GN=C1 PE=2 SV=1 | 65.5 |
| - | 50 | 277 | sp\|Q7VIN2\|NPD_HELHP | NAD-dependent protein deacylase OS=Helicobacter hepaticus (strain ATCC 51449 / 3B1) OX=235279 GN=cobB PE=3 SV=1 | 34.8 |
